# Ancestral ecological regime shapes reaction to food limitation in the Least Killifish, *Heterandria* *formosa*

**DOI:** 10.1002/ece3.7490

**Published:** 2021-04-06

**Authors:** Anja Felmy, Jeff Leips, Joseph Travis

**Affiliations:** ^1^ Department of Zoology University of Oxford Oxford UK; ^2^ Department of Biological Sciences University of Maryland Baltimore County Baltimore MD USA; ^3^ Department of Biological Science Florida State University Tallahassee FL USA

**Keywords:** density‐dependent selection, life history, local adaptation, maternal effects, offspring size, phenotypic plasticity

## Abstract

Populations with different densities often show genetically based differences in life histories. The divergent life histories could be driven by several agents of selection, one of which is variation in per‐capita food levels. Its relationship with population density is complex, as it depends on overall food availability, individual metabolic demand, and food‐independent factors potentially affecting density, such as predation intensity. Here, we present a case study of two populations of a small live‐bearing freshwater fish, one characterized by high density, low predation risk, low overall food availability, and presumably low per‐capita food levels, and the other by low density, high predation risk, high overall food availability, and presumably high per‐capita food levels. Using a laboratory experiment, we examined whether fish from these populations respond differently to food limitation, and whether size at birth, a key trait with respect to density variation in this species, is associated with any such differential responses. While at the lower food level growth was slower, body size smaller, maturation delayed, and survival reduced in both populations, these fitness costs were smaller in fish from the high‐density population. At low food, only 15% of high‐density fish died, compared to 75% of low‐density fish. This difference was much smaller at high food (0% vs. 15% mortality). The increased survival of high‐density fish may, at least partly, be due to their larger size at birth. Moreover, being larger at birth enabled fish to mature relatively early even at the lower food level. We demonstrate that sensitivities to food limitation differ between study populations, consistent with selection for a greater ability to tolerate low per‐capita food availability in the high‐density population. While we cannot preclude other agents of selection from operating in these populations simultaneously, our results suggest that variation in per‐capita food levels is one of those agents.

## Graphical abstract

When populations with contrasting densities have different life histories, one of the agents of selection driving these differences might be variation in per‐capita food levels. We conducted a laboratory experiment to test whether two populations of the Least Killifish, one experiencing a higher and one a lower population density, respond differently to food limitation. As expected if high‐density fish were adapted to food scarcity, we found that they survived significantly better at the lower food level than low‐density fish, suggesting that variation in per‐capita food availability is one of the selective agents that operate in these populations.

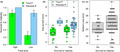

## 1 INTRODUCTION

Ecologists have long been interested in whether animal populations with different densities of conspecifics will evolve adaptive differences in response to these conditions (Berec et al., 2018; Boyce, 1984; Engen & Saether, 2017; Macarthur & Wilson, 1967; Mueller, 1997; Pianka, 1970; Wright et al., 2019). Careful experimental studies of laboratory populations have shown this to be the case (Bull et al., 2006; Mueller, 1988).

One of the challenges in understanding the incidence and importance of density‐dependent evolution is identifying the agents of selection that contribute to adaptation to different population densities. Depending on the selective agents involved, the consequences for population and evolutionary dynamics will be different. For instance, the response to selection can depend on the age classes most affected by density (Charlesworth, 1994; Engen & Saether, 2016; Mueller et al., 2000) as well as the phenotypic traits most readily responsive to differences in population density (Engen et al., 2020; Tung et al., 2019).

The phenotypic divergence between high‐ and low‐density populations can be driven by several distinct agents of selection, depending upon the specific case. Higher density can mean, among other factors, lower per‐capita food levels, a higher rate of stressful social interactions, higher rates of pathogen transmission, greater competition for mates, and, in some systems, higher accumulation rates of waste products (Berec et al., 2018; Than et al., 2020). Laboratory studies have shown that nearly all of these agents can be acting. For example, populations of *Drosophila* kept at high densities show adaptations to food scarcity (Joshi & Mueller, 1988), to a lack of suitable pupation sites (Joshi & Mueller, 1993), to adult crowding (Joshi et al., 1998), and to increased concentrations of urea, a nitrogenous waste product accumulating in crowded cultures (Joshi et al., 1996).

Among the most obvious selection pressures that differ between populations of different density is per‐capita food availability. Laboratory studies have shown that individuals from high‐density populations have adapted to food limitation by increasing their feeding rate (Joshi & Mueller, 1996; Mueller, 1990) and digestive efficiency (Sarangi et al., 2016). In natural populations of Trinidadian guppies (*Poecilia reticulata*), a small freshwater fish, populations at chronically higher densities have an expanded diet compared to populations at chronically lower densities (Bassar et al., 2017; Zandona et al., 2011). Moreover, high‐density populations of guppies have reduced nitrogen excretion rates (El‐Sabaawi et al., 2015), lower resting metabolic rates (Auer et al., 2018), and stoichiometric relations indicative of resource limitation (El‐Sabaawi et al., 2012). These characteristics of high‐density populations correspond with what one might expect as responses to relative food scarcity. Experiments with Hart's killifish (*Rivulus hartii*) have shown that, at low food levels, populations living at consistently higher densities matured earlier and at a smaller size, and produced more eggs, than populations at consistently lower densities (Walsh & Reznick, 2010).

Despite these examples, it cannot simply be assumed that adaptation to lower per‐capita food levels will be evident in all natural populations experiencing higher densities. For one reason, the social environment in a high‐density population can affect survival, growth, and reproduction independently of food levels (Gutierrez et al., 2020; Leatherbury & Travis, 2019) and can be a powerful selective agent in its own right. For another, populations living at different densities need not be experiencing different per‐capita food levels if food availability varies among locations, because the important ecological variable is the ratio of food supply to metabolic demand, and not food level per se (Wilbur, 1977). Finally, abiotic factors may affect the metabolic demand of an organism (Travis & Trexler, 1986; Warner et al., 1991), further complicating the relationships between population density, overall food availability, and per‐capita resource levels. Often experiments are necessary to assess whether high‐density populations indeed experience food scarcity, and, if so, how they have adapted to lower per‐capita food levels.

**FIGURE 1:**
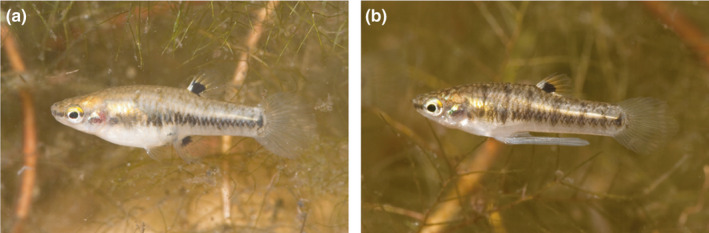
Pregnant female (a) and adult male (b) *Heterandria formosa*. Photograph courtesy of Pierson Hill

The Least Killifish, *Heterandria formosa,* offers an excellent opportunity to investigate this issue because it occurs at vastly different population densities (MacRae & Travis, 2014) and because its small size and external morphological features indicating sexual maturity make it amenable to experimental life‐history studies (Figure 1). *H. formosa* is found throughout the lower coastal plain of the southeastern United States. Populations in north Florida occur in a wide variety of habitats, from freshwater springs to lakes, ponds, and swamps in river bottoms (MacRae & Travis, 2014). In all habitats, *H. formosa* occupies the shallow littoral zone and is a primary consumer (Aresco et al., 2015).

Prior work has shown that population density is dramatically higher and per‐capita predation risk lower at Wacissa River (WR) than Trout Pond (TP; characteristics of both locations summarized in Table 1; Leips & Travis, 1999; MacRae & Travis, 2014; Richardson et al., 2006). These ecological differences co‐occur with differences in the life history of *H. formosa*: In WR, the high‐density/low‐predation population, size at maturity is larger, fecundity lower, and offspring size at birth larger compared with fish from TP (references in Table 1).

**TABLE 1:** Characteristics of study populations

	Trout Pond (TP)	Wacissa River (WR)	References
Ecological variables			
Population density	Lower	Higher	1–3
Predation regime	Higher	Lower	1–3
Primary productivity	Likely higher	Likely lower	This study
Phenotypic variables in *H. formosa*			
Size at birth	Smaller	Larger	1, 4–9, this study
Size at maturity	Smaller	Larger	4, 6, 10, this study
Age at maturity	No difference	No difference	4, 11, this study
Survival to maturity	Lower	Higher	This study
Fecundity	Higher	Lower	1, 6, 7

Not<del author="Anja Felmy" command="Delete" timestamp="1617025334808" title="Deleted by Anja Felmy on 29.3.2021, 14:42:14" class="reU3" id="edit4">e</del>

References: 1 (Leips & Travis, 1999), 2 (MacRae & Travis, 2014), 3 (Richardson et al., 2006), 4 (Hale & Travis, 2015), 5 (Leips et al., 2009), 6 (Leips et al., 2000), 7 (Schrader & Travis, 2005), 8 (Schrader & Travis, 2008), 9 (Schrader & Travis, 2012), 10 (Landy & Travis, 2018), 11 (Leips et al., 2013).

The divergent life histories could be the product of several, nonexclusive factors. First, the two locations have many abiotic differences. TP is a small (5 ha), soft‐water, acidic lake, with a conductivity between 12 and 25 μMHOS, a pH between 4.6 and 5.3, and water temperatures fluctuating by 25°C between summer and winter. By contrast, WR is a spring‐fed, hard‐water river with a pH of 7.1 to 8.4, a conductivity between 150 and 275 μMHOS, and weaker fluctuations in water temperature of up to 11°C between seasons (Leips & Travis, 1999). However, a laboratory study in which *H. formosa* from both locations were reared either in water from TP or spring water found no evidence that the two populations differ in their responses to water chemistry (Hale & Travis, 2015).

Among the biotic differences between TP and WR, their strikingly different population densities and predation rates have received most attention. Although this contrast is particularly pronounced in TP and WR, higher population densities are associated with lower per‐capita predation risks, and *vice versa*, across many populations of *H. formosa* (MacRae & Travis, 2014). Differences in density and predation co‐occur with specific aspects of the phenotype. Across nine populations, including TP and WR, both higher densities and lower risks of predation were associated with larger offspring (Schrader & Travis, 2012), while density and predation risk were associated with different aspects of male body shape (Landy & Travis, 2015).

Density‐manipulation experiments in the laboratory have tested whether life‐history traits were less affected by high density in fish from high‐density origins than in fish from low‐density origins, suggestive of local adaptation. Results appeared to depend on whether density treatments were allowed to create different per‐capita food levels. When density was manipulated but per‐capita food levels held constant, TP and WR fish did not differ in their response to density: the depressant effects of density on reproductive traits (Leips et al., 2009) and on somatic growth, age at maturity, and size at maturity (Leips et al., 2013) were similar in individuals from TP and WR. In contrast, when density was manipulated without holding food levels constant, some life‐history traits were found to respond differently to density in both populations, with offspring size at birth being reduced much more in WR fish but brood size reduced much more in TP fish when exposed to the same high density (Leips et al., 2000). Intriguingly, the degree of sensitivity in the latter study, which also manipulated the genetic composition of experimental populations, was proportional to the initial dosage of WR alleles, showing that population differences have a genetic component (Leips et al., 2000).

**FIGURE 2:**
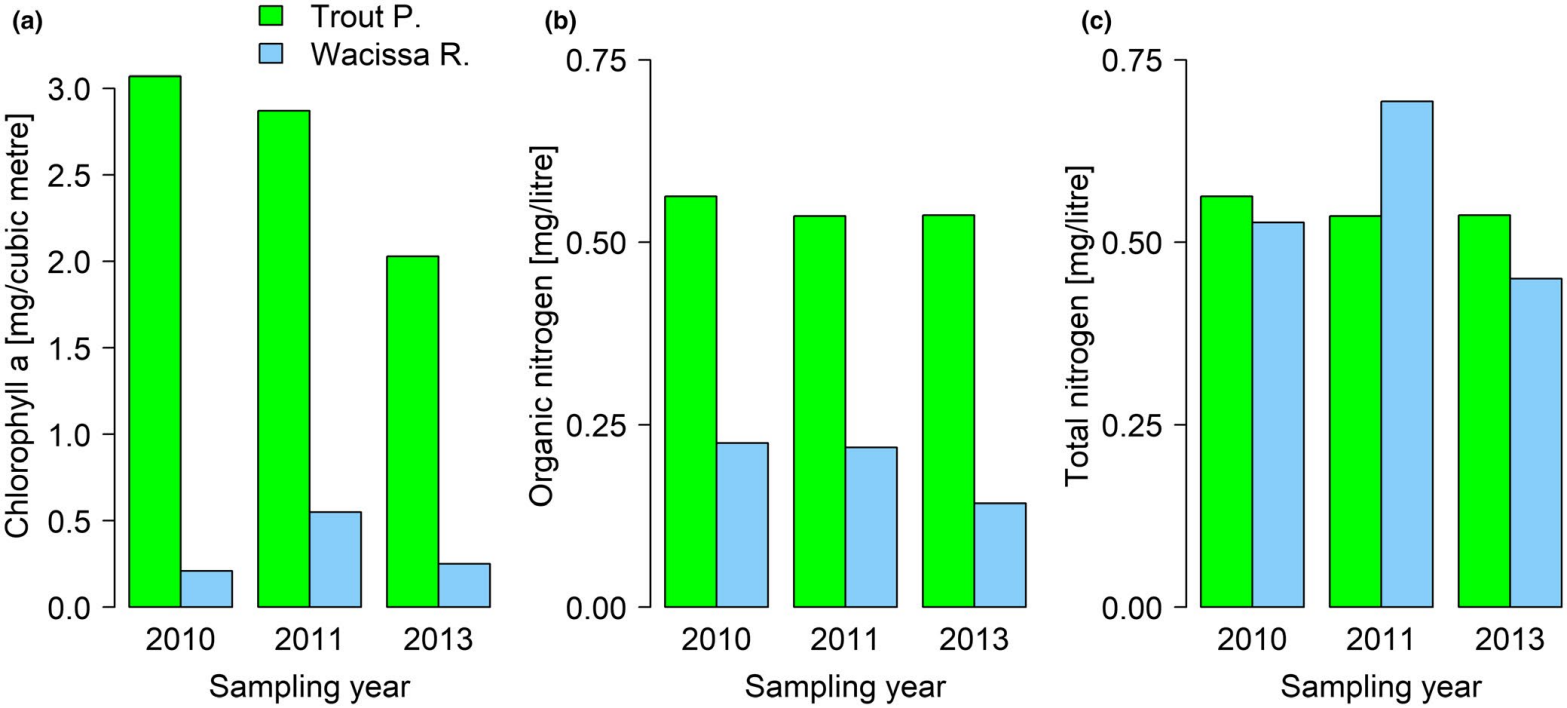
Differences in the concentration of chlorophyll a (a), organic nitrogen (b), and total nitrogen (c) in water from Trout Pond and Wacissa River across three years. In TP, concentrations of nitrate and nitrite (i.e., inorganic nitrogen) were below the detection limit, indicating that these were taken up rapidly by algae and aquatic plants. In WR, nitrite concentrations were also undetectable, but nitrate concentrations were substantial (i.e., total nitrogen minus organic nitrogen). Total phosphorus values were below the detection limit of 0.014 mg/L for both water bodies

Per‐capita food availability may be higher in TP than WR not only as a consequence of lower density in TP; TP also likely has an increased primary productivity. This was suggested by an analysis of water samples taken from either location in 2010, 2011, and 2013, which showed that, in TP, concentrations of chlorophyll a were 5.2 to 14.6 times higher (Figure 2a), and concentrations of organic nitrogen 2.5 to 3.8 times higher, than in WR (Figure 2b). Furthermore, in TP all of the nitrogen was organic (total/organic N ratio = 1.0), whereas in WR nitrogen predominantly occurred as inorganic nitrate (total/organic N ratio = 2.3–3.2, Figure 2c), indicating that in TP inorganic nitrogen is taken up rapidly by algae and aquatic plants. Although these data reflect standing crop and not productivity per se, they strongly suggest that TP is the more productive habitat.

To date, it is unclear to what extent the differential sensitivity to density in TP and WR fish reflects an adaptation to presumably lower per‐capita food levels in WR than TP, potentially leading to food scarcity in WR. Although TP and WR fish have, separately, been used in food‐manipulation experiments in the past (Leatherbury & Travis, 2019; Travis et al., 1987), the populations’ responses to food limitation have never been compared directly. In this case study, we therefore tested the hypothesis that fish from WR were indeed better at surviving to maturity and maintaining somatic growth in the face of food scarcity than fish from TP, as expected if WR fish were adapted to lower per‐capita food availability. Furthermore, both correlational field studies (Leips & Travis, 1999; Schrader & Travis, 2005, 2012) and laboratory experiments (Hale & Travis, 2015; Leips et al., 2000, 2009; Schrader & Travis, 2008) have identified offspring size at birth as a key trait with regard to density variation. We therefore tested whether a larger size at birth was associated with higher survival, larger size at maturity, and earlier maturation, and, where possible, how these relationships depended on food levels.

We subjected second‐generation laboratory‐reared individuals from both populations to one of two food levels and measured seven life‐history traits: size at birth, at 14 days, at 28 days, at 42 days, and at sexual maturity, as well as survival to and age at maturity. Altogether, we found that WR fish survived significantly better at the lower food level than TP fish, in agreement with an increased ability of WR fish to deal with food limitation. A larger size at birth increased survival, likely in part explaining the higher survival of food‐limited WR fish. Moreover, a larger size at birth allowed fish to mature relatively early even at the lower food level.

## 2 MATERIALS AND METHODS

### 2.1 Study species

*Heterandria formosa* is a small poeciliid fish inhabiting ponds and streams in the coastal plain of the southeastern United States. Male standard length (SL, the distance from the tip of the snout to the hypural plate in the tail) varies, typically, from 10 mm to 25 mm and female SL from 9 to 15 mm. Reproduction is placental and matrotrophic; females provide almost all nourishment for their embryos after fertilization (Schrader & Travis, 2005). Females carry several temporally overlapping broods, a phenomenon called superfetation (Travis et al., 1987). Offspring are born precocial. Females reach sexual maturity after 40 to 65 days and males after 50 to 90 days (Hale & Travis, 2015). After a gestation period of 25 to 28 days, females give birth to small broods (average brood size 2.5 to 2.9 offspring) at intervals of 12–16 days (Travis et al., 1987).

### 2.2 Measurements of water quality

Water samples were collected from Trout Pond (TP), Wakulla County, Florida, and from Wacissa River (WR), Jefferson County, Florida, in 2010, 2011, and 2013, by immersing a sterile, pint‐sized Nalgene bottle in water. A second sterile Nalgene bottle was filled with water poured through a funnel and filter paper. Both bottles were filled to capacity to avoid trapped air and were then sealed, placed on ice, and directly delivered to Ackuritlabs, Inc. The data were part of a larger survey of lakes and springs near Tallahassee carried out in those three years. Ackuritlabs, Inc. measured the concentration of total nitrogen, organic nitrogen, nitrate, nitrite, total phosphorus, and chlorophyll a. They ran blanks and duplicates as part of their standard quality control process.

### 2.3 Experimental design

We collected adults from TP and from WR in September 1993 and used them to establish breeding colonies in the laboratory. We housed the adults from each population in two 76‐L aquaria per population at a density of 4–5 males and 4–5 females per tank and collected F1 offspring as they were born. Those offspring were raised in 38‐liter aquaria until maturity, when we removed females and mated them to males from different aquaria to minimize inbreeding. We isolated pairs in small aquaria (4 L) and inspected them daily for the presence of newborn F2 offspring; these were used in our experiment. The first F2 offspring were born in February 1994. Within 24 hr of their birth, F2 offspring were moved to plastic containers (750 ml) in which they were individually housed for the whole duration of the experiment.

All aquaria were filled with well water and aerated continuously with airline tubes. Experimental tanks were kept at a constant temperature of 29°C on a 14:10‐h light:dark cycle under aquarium lamps at Florida State University. Twice a week, two thirds of the water in each tank was replaced with fresh water, and tanks were cleaned once per week. Every second week a randomly chosen tank was tested for appropriate pH, ammonia, nitrite, and nitrate levels. Water quality was good throughout the experiment and did not differ between experimental groups.

The experiment was a factorial design in which we raised offspring to maturity from either population (TP or WR) at one of two food levels, designated “high” or “low.” All fish were fed ground TetraMin fish flakes. We set the initial “high” food ration as 1 mg/day and “low” as 0.25 mg/day. We increased food rations at day 12, 24, 42, and 63 to accommodate growth, up to a maximum at day 63 of 20 mg/day at “high” and 5 mg/day at “low.” “Low” food was always set as one‐quarter of “high.” We based these food levels on extensive preliminary rearings performed in 1983. At high food levels, some uneaten food was usually found at the next feeding (at which point it was removed), while at low food levels, all the food was consumed shortly after it was added. Earlier work showed that, for WR, the range of female body sizes when using these food levels represents the size distribution in the natural population in late summer (Travis et al., 1987). Field‐caught and F1 fish were fed to satiation.

In total, our experiment included 80 individuals: 20 each per population and food level (see Table 2 for details). WR fish came from 16 and TP fish from 15 F1 mothers, with an average of 2.6 offspring per mother (range 1–5). For each mother, we used offspring from a single brood and split them between food levels. However, brood size is small in *H. formosa* (average 2.5 to 2.9 offspring per brood; Travis et al., 1987). Six mothers were thus represented by a single offspring. By using F2 individuals, keeping fish in a common environment, feeding them controlled amounts of food, and splitting pairs of full‐siblings between food levels if possible, we minimized maternal, environmental, and other nonheritable sources of variation.

**TABLE 2:** Number of focal individuals as a function of population of origin, experimental food level, survival to sexual maturity, and sex

Population	Treatment	Initial sample size	Number of Survivors		Number of Deaths
			Male	Female	Sex unknown
Trout Pond (TP)	High food	20	9	8	3
	Low food	20	0	5	15
Wacissa River (WR)	High food	20	12 (11)	8	0
	Low food	20	10	7	3

Note

Only fish that survived could be sexed. Note that one surviving male from WR assigned to the higher food level was excluded from most statistical analyses due to a potential measurement error, bringing the sample size down to 11 in that group.

### 2.4 Measurement of life‐history traits

We measured the standard length (SL) of each individual F2 fish at birth, at 14 days, 28 days, 42 days, and at sexual maturity. Additionally, we measured the SL of F1 mothers when they reached maturity and were put into individual aquaria with a male to start breeding. To measure each F2 individual's SL, we removed the fish from its aquarium, placed it in a small petri dish against a millimeter scale, photographed it, and returned it to its aquarium. Lengths were then manually read from the photographs. No individual died as a result of this procedure. Photographs were taken using a Canon single‐lens reflex camera with a 55‐mm macro lens. Photographic length measurements were very precise (repeatability *r* = .99, effect of individual identity in a simple ANOVA: *F*_36,37_ = 381.3, *p* < .0001, *n* = 37 individuals that were photographed and measured twice). We also estimated the strength of the correlation between standard length and dry mass by measuring 20 fry at birth using both methods. The standard length of newborn fry was measured by photographing them as described above. Fry were then euthanized in MS‐222, preserved in formalin, dried under vacuum for 72 hr (60°C and −54.7 kPA), and weighed to the nearest 0.01 mg. Photographic length measurements and dry weights were strongly correlated (Pearson's product‐moment correlation: *r* = .76, *t*_18_ = 5.01, *p* < .0001). F1 mothers were measured by placing them in a petri dish into which a millimeter grid was laminated.

Additional traits included in this study are the survival to sexual maturity and the age when individuals attained maturity. Each F2 individual was inspected daily throughout the experiment to monitor survival and to assess its stage of maturation. A male was considered to be mature when his gonopodium (modified anal fin used as an intromittent organ) was fully formed; a female, when the characteristic black spot appeared on her anal fin. Fish were sexed on the day when they attained sexual maturity.

The body sizes and age at maturity of one individual, a surviving male from WR kept at the higher food level, may contain measurement errors. We thus excluded this individual from all analyses except that of survival to maturity, where the individual was retained but its size at birth was not used as a covariate in the analysis.

### 2.5 Statistical analysis

We used generalized linear mixed models to analyze size at birth, survival to sexual maturity, size at 14 days, and age at maturity, and an analysis of variance with repeated measures to jointly analyze size at 14 days, at 28 days, at 42 days, and at maturity. Details of all models are provided in Table 3, including the type of model fitted, the list of predictors used, how many individuals were excluded from each model, why these individuals were excluded, and each model's sample size.

**TABLE 3:** Traits considered in this study and details of how they were modeled

Trait	Model	Predictors	Excluded individuals	Sample size
Size at birth	GLMM, Gaussian errors	Fixed: POP, FOOD, MATSZ, POP × MATSZ Random: MATID	Fish H7	79
			Fish H7, 3 outliers (size at birth >8 mm)	76
		Fixed: POP, FOOD, MATSZ, POP × MATSZ, SEX Random: MATID	Fish H7, 21 fish with unknown sex	58
			Fish H7, 21 fish with unknown sex, 3 outliers (size at birth >8.0 mm)	55
Survival to maturity	GLMM, binomial errors	Fixed: POP, FOOD, SBIR, MATSZ Random: MATID	–	80
		Fixed: FOOD, SBIR, MATSZ Random: MATID	–	80
Size at 14 days	GLMM, Gaussian errors	Fixed: POP, FOOD, POP × FOOD, SBIR, MATSZ Random: MATID	Fish H7, 13 fish that did not survive, 1 fish with missing data	65
			Fish H7, 13 fish that did not survive, 1 fish with missing data, 4 outliers (size at 14 days > 9 mm)	61
Juvenile sizes and size at maturity	ANOVA with repeated measures	POP, FOOD, AGE, POP × AGE, FOOD × AGE, SEX, SEX × AGE, SBIR, SBIR × AGE, MATSZ, MATID Error term: INDID	Fish H7, 21 fish that did not survive, 4 fish with missing data	216 (i.e., 54 fish × 4 traits)
Age at maturity	GLMM, Gaussian errors	Fixed: POP, FOOD, SBIR, FOOD × SBIR, SEX Random: MATID	Fish H7, 21 fish that did not survive, 2 fish with missing data	56
			Fish H7, 21 fish that did not survive, 2 fish with missing data, 3 outliers (2 with age at maturity < 36 days, 1 with age at maturity > 85 days)	53

Note

Size at birth, size at 14 days, and age at maturity were analyzed using GLMMs with Gaussian errors because they were approximately normally distributed, as judged from quantile–quantile plots (QQ plots) and two‐sided Kolmogorov–Smirnov tests (size at birth: *D* = 0.08, *p* = .70; size at 14 days: *D* = 0.12, *p* = .34; age at maturity: *D* = 0.11, *p* = .49). Fish H7 was excluded from most models because its size and age data may contain measurement errors.

Abbreviations: AGE, age category (i.e., at 14 days, at 28 days, at 42 days, at maturity); FOOD, experimental food levels; GLMM, generalized linear mixed model; INDID, individual identity; MATID, maternal identity; MATSZ, maternal size; POP, population identity; SBIR, size at birth; SEX, sex.

The list of predictors varied among models, but always included the population of origin (Trout Pond vs. Wacissa River) and the experimental food level (high vs. low). Unfortunately, we could only formally test for an interaction between population and food level in one of the four traits (size at 14 days) for which that test would have been interesting; for size at birth the interaction was meaningless, as the food treatment had not yet begun and was merely included as a predictor to test for a potential assignment bias. The reason for our inability to fit the interaction differed among traits, but was due to the differential survival rates of TP and WR fish at high and low food availability. For age at maturity and the repeated‐measures ANOVA of growth to maturity, the problem was the low number of fish from TP kept at the lower food level that survived to maturity (*n* = 5); note that the ANOVA required nonmissing data from each individual for each of the four measured sizes. Conversely, for survival to maturity, it was the lack of dead fish from WR that were kept at the lower food level (*n* = 0) which prohibited us to include the interaction in the model, compounded by the fact that the main effects of population (92.5% surviving fish from WR vs. 55.5% from TP) and of the food treatment (92.5% surviving fish at high food vs. 55.5% at low food) were exactly identical. We therefore used a chi‐square test, which proved robust to the mirror‐image effects of population and food level, to compare the number of dead and surviving fish among the four experimental groups. To identify the cells in the contingency table that accounted for most of the difference between expected and observed values, we computed the relative contribution of each cell to the total chi‐square score as $100*(r^{2}/\chi_{3}^{2})$, where *r* was the Pearson residual and $\chi_{3}^{2}$ the chi‐square score.

The differential survival of fish in the four experimental groups also complicated the inclusion of other predictors in some of our models. It is not possible to determine the sex of a juvenile from external morphology and so the sex of fish that died before attaining sexual maturity remains unknown. Sex as a categorical predictor with three levels (female, male, unknown) was thus confounded with both population identity and food level (i.e., 15/21 individuals with unknown sex being low‐food fish TP). For size at birth, we circumvented the problem by testing for a size difference between males and females in a separate model using only the individuals of known sex. For size at 14 days, we decided against using the same approach to prevent having insufficient statistical power due to a reduced sample size (*n* = 57 fish of known sex and size at 14 days). For survival, the exclusion of nonsurviving individuals was no option, and so sex differences in survival could not be investigated. For age at maturity and the repeated‐measures ANOVA, the inclusion of sex was unproblematic. Lastly, when analyzing survival, we could not test the interaction between food level and size at birth (*n* = 3 fish that died at the higher food level), nor the interaction between population identity and size at birth (*n* = 3 fish from WR that died).

All GLMMs included maternal identity as a random effect. We tested its significance with log‐likelihood ratio tests comparing the full model to one without maternal identity. The repeated‐measures ANOVA contained individual identity as the single error term, to account for repeated measures of fish. The error term specified two error strata, with appropriate models fitted within each stratum. We tested the effects of age and all its interactions within individuals, while we tested all the other effects between individuals.

Some models initially contained predictors that were dropped from the final model because they had no impact on the response. For size at 14 days, these dropped predictors include the interactions between food level and size at birth, between population and size at birth, and between population and maternal size (elimination criterion: ratio of $\chi^{2}$ over its degrees of freedom < 1 in log‐likelihood ratio tests comparing a model with and without the interaction in question). In the repeated‐measures ANOVA, we dropped the interactions between age and maternal size, between population and size at birth, between food level and sex, and between population and sex (elimination criterion: *F*‐value < 1). To ensure sufficient statistical power in our models of age at sexual maturity despite the reduced sample size (*n* = 56 fish with known age at maturity), we did not include nonexperimental (i.e., purely correlative) predictors that, upon visual inspection, appeared unrelated to the age at sexual maturity (e.g., maternal size, size at maturity, and the interaction between size at birth and population). In addition, we could not fit the interactions between population and sex, and food level and sex, because in each of these cases one or several treatment levels had insufficient sample sizes (i.e., < 10 data points).

For size at birth, size at 14 days, and age at maturity, quantile–quantile plots (QQ plots) identified a small number of data points as potential outliers. We have no reason to believe that these outlying points represent measurement errors, but to assess whether they drive the patterns we see, we fitted additional models after excluding them.

We performed all statistical analyses in R 4.0.0 (R Core Team, 2020). We fitted GLMMs using function “glmmTMB” in R‐package “glmmTMB” (Brooks et al., 2017), and the repeated‐measures analysis of variance using function “aov” (R Core Team, 2020). We assessed the fit of all models that converged with diagnostic plots. The reference levels for categorical predictors were Trout Pond, high food, female. Values are given as mean ± *SD*. Binomial standard errors for survival rates were computed according to Zar (1996) as $SE=\sqrt{\left(p(1‐p))/(n‐1\right)}$, where p was the proportion of fish that survived and n the total number of fish. Scatter plots were prepared using R‐package “beeswarm” (Eklund, 2016) and depict individual data points superimposed on boxplots. The entire code and model output, prepared using R Markdown (Allaire et al., 2020; Xie et al., 2018), can be found in the Appendix S1.

## 3 RESULTS

### 3.1 Size at birth

**FIGURE 3:**
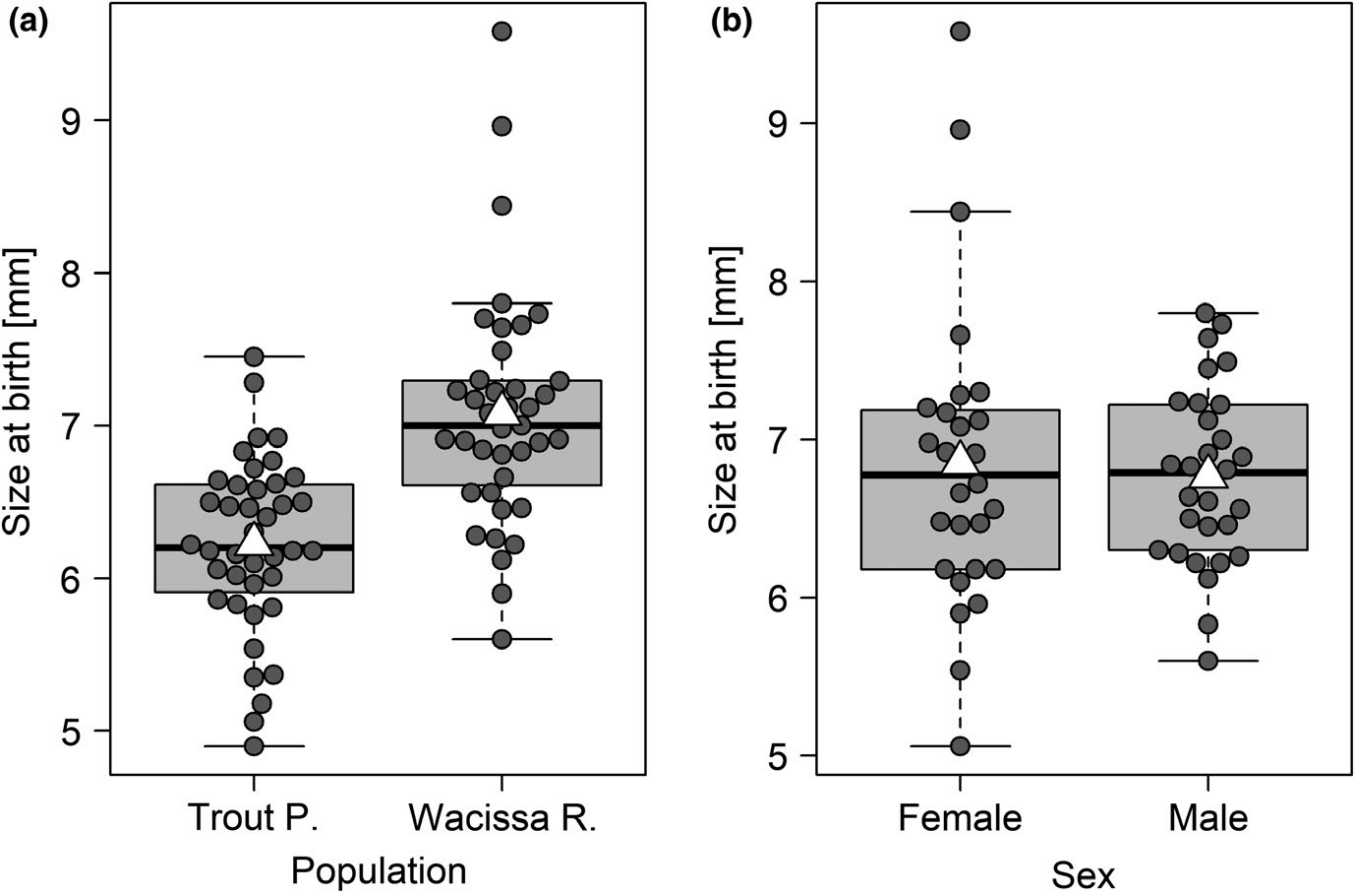
Size at birth was affected by population of origin (a), but did not differ between males and females (b). White triangles on boxplots show group means. In (b), fish of unknown sex are excluded

Fish from Wacissa River, the low‐predation/high‐density location, were about 15% larger at birth than fish from Trout Pond (partial regression coefficient *b* = 3.81, *z* statistic = 2.31, *p* = .0209, Figure 3a). They were also slightly larger when assigned to the high‐ rather than the low‐food treatment, although this effect is coincidental, as fish were measured before they were fed for the first time (*b* = −0.31, *z* = −2.73, *p* = .0063). There was no overall effect of maternal size on offspring size at birth (*b* = 0.09, *z* = 1.61, *p* = .11), and no evidence for an interaction between maternal size and population (*b* = −0.13, *z* = −1.84, *p* = .07). Maternal identity, included as a random effect, explained a significant amount of variation in offspring size at birth ($\chi_{1}^{2}$ = 9.45, *p* = .0021). We tested for a size difference between the sexes using only the subset of fish with known sex and found that males and females did not differ in size at birth (*b* = −0.26, *z* = −1.85, *p* = .06, Figure 3b). The inclusion of sex as a predictor left the remaining model results largely unchanged (data not shown).

Some of these results were contingent on three exceptionally large fry (> 8.0 mm), all of them from WR and assigned to the higher food level. When we excluded these fry, the population of origin still had a strong effect (*b* = 2.94, *z* = 2.23, *p* = .0258, Figure 3a), but the coincidental effect of food level disappeared (*b* = −0.17, *z* = −1.53, *p* = .13). Moreover, the main effect of maternal size became significant, indicating that larger mothers were likely to produce larger offspring (*b* = 0.10, *z* = 2.12, *p* = .0340). The interaction between maternal size and population remained nonsignificant (*b* = −0.10, *z* = −1.73, *p* = .08). When using the outlier‐free dataset, maternal identity, independent of maternal size, did not predict size at birth ($\chi_{1}^{2}$ = 2.36, *p* = .12). The size difference between males and females remained nonsignificant (*b* = −0.02, *z* = −0.16, *p* = .88, Figure 3b).

### 3.2 Survival to maturity

**FIGURE 4:**
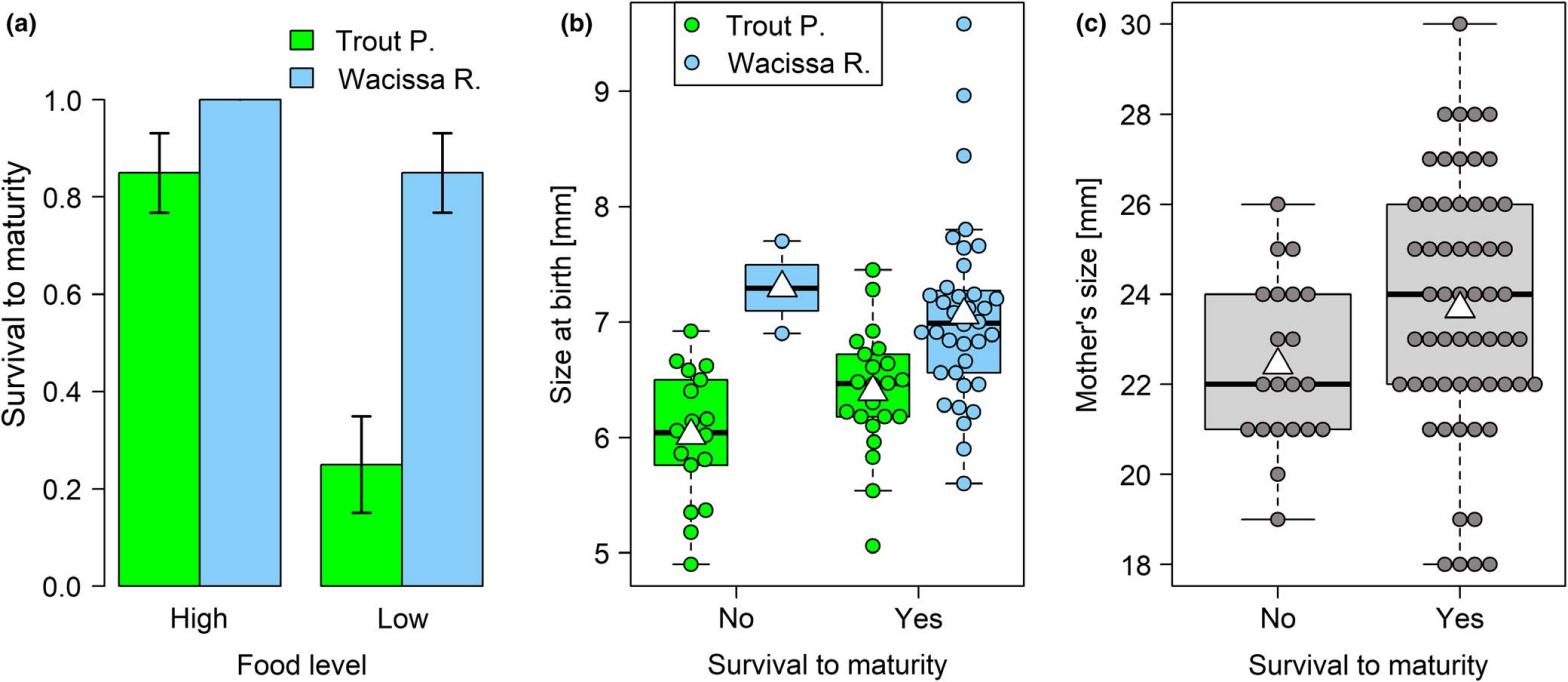
Survival was reduced at the lower food level, particularly in fish from Trout Pond (a), and positively associated with size at birth (b) but not maternal size (c). In (a), sample size is 20 fish for each vertical bar, and error bars are binomial standard errors (±1 *SE*). Note that survival was 100% for fish from Wacissa River kept at high food, and the binomial standard error consequently zero. White triangles on boxplots show group means

Twenty‐six percent of fish died before reaching sexual maturity. The mortality rate was highest in the two weeks after birth (16.3%), intermediate between 14 and 28 days of age (7.5%), and lowest between 28 days and the age when fish attained maturity (2.5%). Survival to maturity was significantly higher among fish from WR (92.5%) than among fish from TP (55.0%, *b* = 2.95, *z* = 2.91, *p* = .0037, Figure 4a). An effect of the same exact size was caused by the food treatment, with 92.5% surviving fish under high‐ and only 55.5% under low‐food conditions (*b* = −2.94, *z* = −3.57, *p* = .0004, Figure 4a). The association between size at birth and survival was nonsignificant (*b* = 0.16, *z* = 0.26, *p* = .79). However, as shown earlier, fish from WR were significantly larger at birth (Figure 3a), causing the predictors “population identity” and “size at birth” to be nonindependent. Accordingly, once population identity was removed from the list of predictors, a larger size at birth was associated with increased survival (*b* = 1.22, *z* = 2.54, *p* = .0111, Figure 4b). Taken together, these findings suggest that the higher survival rate of fish from WR may, in part, be due to their larger size at birth. Survival did not depend on maternal size (*b* = 0.28, *z* = 1.67, *p* = .10, Figure 4c), and maternal identity did not explain any variation in offspring survival ($\chi_{1}^{2}$ = 0.00, *p* = 1.00).

Fish from TP were more sensitive to food limitation than fish from WR: at the lower food level, 75% of fish from TP died, compared to only 15% of fish from WR (Figure 4a). Mortality rates were much lower at the higher food level and not so different between populations: 15% in TP and 0% in WR. This population identity × food level interaction was evident in a chi‐square test ($\chi_{3}^{2}$ = 34.29, *p* < .0001), of which the residuals showed an excess of dead (Pearson residual *r* = 4.26) and concomitant lack of surviving fish (*r* = −2.54) that were from TP and kept at the lower food level, and a lack of dead fish that were from WR and kept at the higher food level (*r* = −2.29). Accordingly, the cells in the contingency table that contributed the most to the total chi‐square score were dead fish/TP/low‐food (52.8%), surviving fish/TP/high‐food (18.8%), and dead fish/WR/high‐food (15.3%). Together, these cells accounted for 86.9% of the observed difference between expected and observed frequencies.

### 3.3 Size as a juvenile and at maturity

**FIGURE 5:**
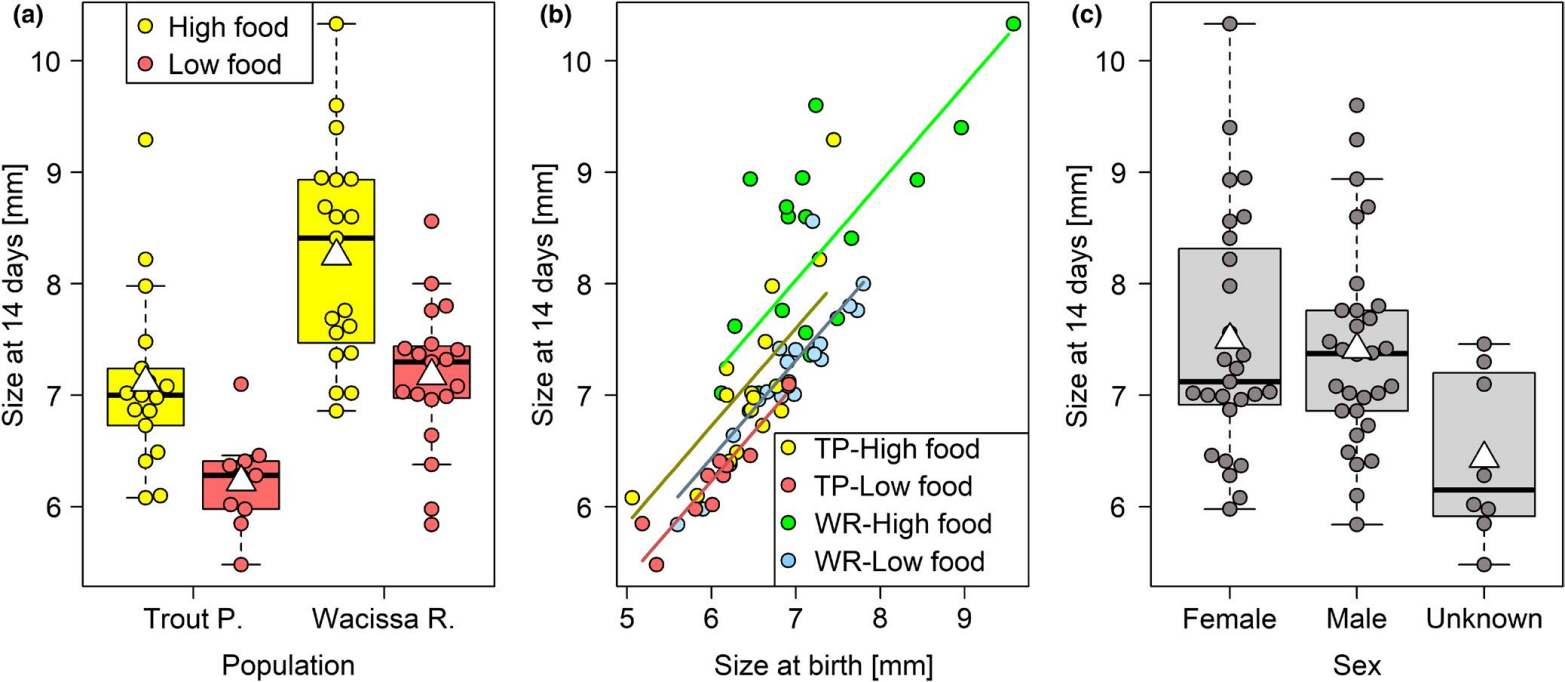
Size at 14 days was reduced at the lower food level and in fish from Trout Pond (a), was highly correlated to size at birth (b) and potentially smaller in fish that did not reach maturity (c). The relationship between size at 14 days and sex (c) is shown for illustrative purposes only, as the low number of fish that did not survive to sexual maturity and hence could not be sexed (*n* = 8) precluded its inclusion in the statistical model. White triangles on boxplots show group means

Fish from WR were about 14% larger than fish from TP at 14 days of age (*b* = 0.42, *z* = 2.24, *p* = .0248), and fish on the high food ration were about 13% larger at this age than fish on the low food ration (*b* = −0.49, *z* = −2.76, *p* = .0058, Figure 5a). There was no interaction between population and food level (*b* = −0.20, *z* = −0.92, *p* = .36, Figure 5a). At that young age, an individual's size was still strongly correlated with its size at birth (*b* = 0.88, *z* = 9.03, *p* < .0001, Figure 5b) and was positively associated with maternal size (*b* = 0.05, *z* = 2.05, *p* = .0400). The effect of maternal identity was nonsignificant ($\chi_{1}^{2}$ = 2.00, *p* = .16). Sex could not be included in the model, but a visual inspection of the data tentatively suggests that males and females did not differ in size at age 14 days, while fish that died as juveniles may potentially have been relatively small (Figure 5c).

When excluding the four largest fish (> 9 mm; three from WR, one from TP, all high‐food; including two that were also exceptionally large fry), identified as potential outliers, the influence of food level grew stronger (*b* = −0.50, *z* = −3.46, *p* =.0005) and the effect of maternal identity became significant ($\chi_{1}^{2}$ = 8.30, *p* =.0040). The correlation with maternal size disappeared (*b* = 0.03, *z* = 0.84, *p* =.40). The other model results remained largely unchanged (population: *b* = 0.46, *z* = 2.52, *p* = .0116; population‐food‐level interaction: *b* = −0.09, *z* = −0.51, *p* = .61; size at birth: *b* = 0.75, *z* = 7.59, *p* < .0001).

**FIGURE 6:**
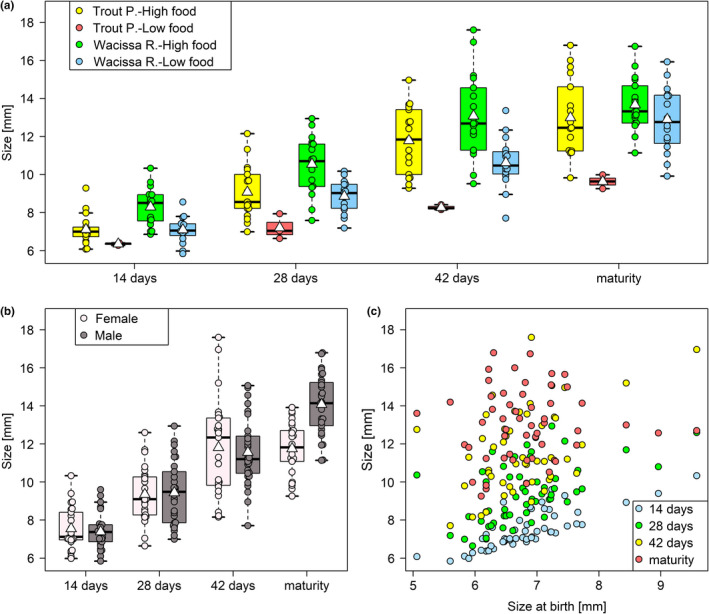
Body size was reduced at the lower food level and in fish from Trout Pond (a), as well as in mature females (b), and its relationship with size at birth gradually disappeared as fish grew older (c). This analysis only included fish that reached sexual maturity and were measured on all four occasions (*n* = 54). White triangles on boxplots show group means

A joint analysis of all juvenile sizes and size at sexual maturity (model results in Table 4) showed that fish from TP were always smaller (significant main effect of population), even at maturity, yet did not grow more slowly than fish from WR (nonsignificant age × population interaction, Figure 6a), suggesting that population differences in juvenile size stemmed from the differential size at birth. Fish did grow more slowly under low‐food conditions (significant main effect of food, and age × food interaction, Figure 6a). The overall effect of sex was nonsignificant, owing to the fact that males and females did not visibly differ in size at 14, 28, and 42 days of age, but once they reached maturity males were considerably larger than females (significant age × sex interaction, Figure 6b). Juvenile size also showed a positive statistical association with size at birth, which was most pronounced at 14 days and gradually disappeared at older ages, until no effect was left at sexual maturity (significant main effect of size at birth, and age × size‐at‐birth interaction, Figure 6c). Neither maternal size nor maternal identity had a bearing on somatic growth (Table 4).

**TABLE 4:** Analysis of variance with repeated measures of juvenile sizes and size at sexual maturity

	*df*	Sum Sq	Mean Sq	*F*‐value	*p*‐value
Error: between individuals					
Population	1	31.75	31.75	9.84	*.0045*
Food level	1	130.84	130.84	40.55	*<.0001*
Sex	1	1.63	1.63	0.51	.48
Size at birth	1	31.60	31.60	9.79	*.0046*
Maternal size	1	10.11	10.11	3.13	.09
Maternal identity	24	89.34	3.72	1.15	.36
Residuals	24	77.45	3.23		
Error: within individuals					
Age	3	974.32	324.77	325.05	*<.0001*
Age × Population	3	0.58	0.19	0.19	.90
Age × Food level	3	16.19	5.40	5.40	*.0015*
Age × Sex	3	66.59	22.20	22.22	*<.0001*
Age × Size at birth	3	10.46	3.49	3.49	*.0174*
Residuals	147	146.87	1.00		

Note

The analysis includes standard lengths measured at 14 days, 28 days, 42 days, and at sexual maturity. Only individuals that survived to maturity and were measured on all four occasions were included (*n* = 54). Standard length was measured in mm. Italics: *p* < .05.

Abbreviations: *df*, degrees of freedom; Mean Sq, mean squares; *SD*, standard deviation; Sum Sq, sum of squares.

### 3.4 Age at sexual maturity

**FIGURE 7:**
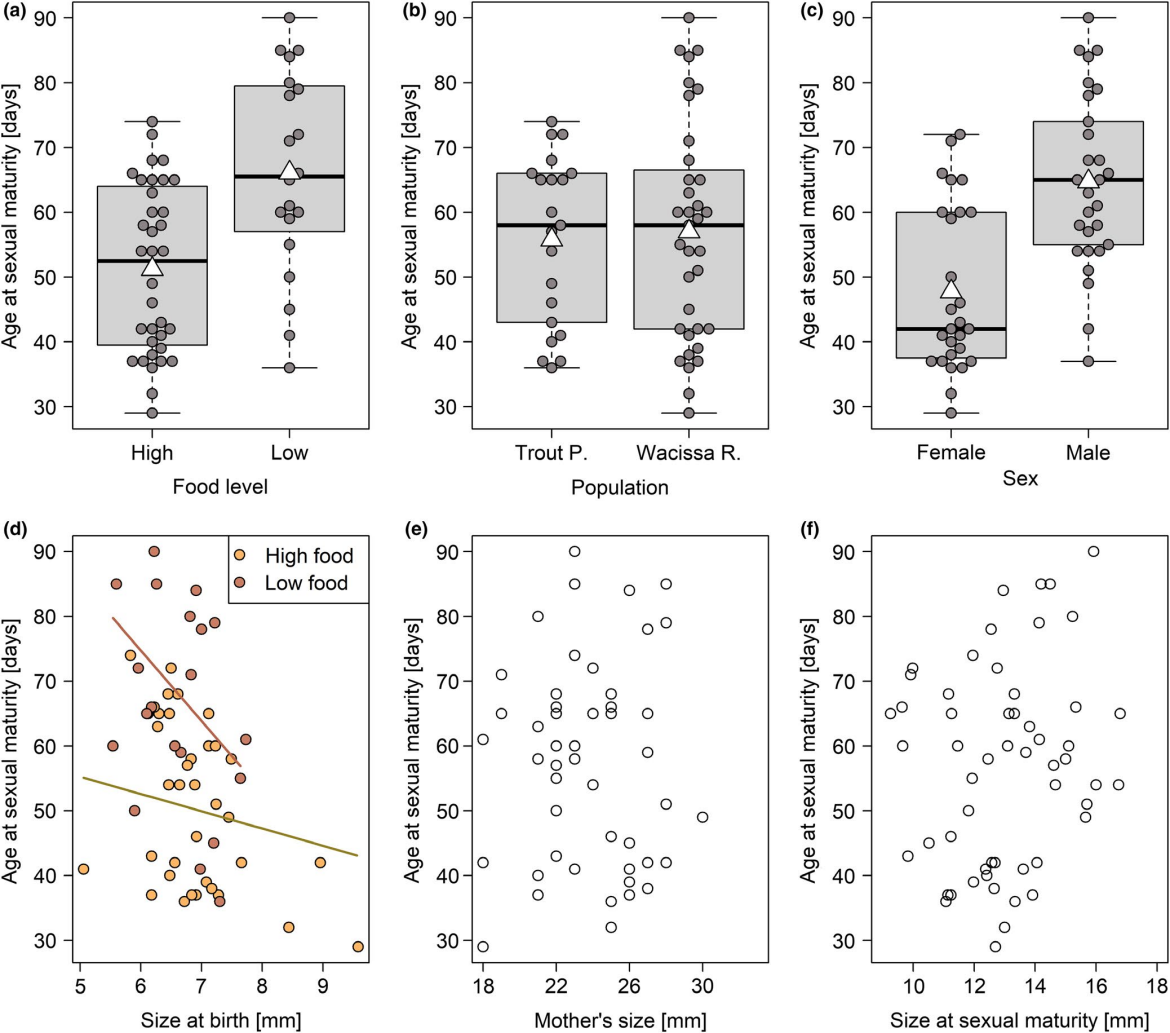
The age at sexual maturity was later at the lower food level (a), especially in fish that were small at birth (d), and was earlier in females than in males (c), but did not differ between study populations (b) and was uncorrelated with maternal size (e) and size at maturity (f). The relationships of age at maturity with maternal size (e) and with size at maturity (f) are shown for illustrative purposes only, and were not included in the statistical model. White triangles on boxplots show group means

Fish attained sexual maturity about 29% later when fed a low‐quantity diet (*b* = 71.50, *z* = 2.87, *p* = .0042, Figure 7a), but fish from the two populations did not differ from each other in age at maturity (*b* = −2.65, *z* = −0.91, *p* = .37, Figure 7b). The interaction between population and food level could not be analyzed statistically due to the poor survival of fish from TP under low‐food conditions (*n* = 4). On average, males matured substantially later than females (65 ± 13 days vs. 48 ± 13 days: *b* = 19.64, *z* = 7.85, *p* < .0001, Figure 7c). Although there was no main effect of size at birth (*b* = −2.66, *z* = −1.32, *p* = .19), its interaction with the feeding regime was significant (*b* = −8.22, *z* = −2.23, *p* = .0260, Figure 7d), indicating that fish kept at the lower food level reached sexual maturity earlier when they were born relatively large. Maternal identity did not explain any variation ($\chi_{1}^{2}$ = 0.00, *p* = 1.00). The exclusion of two females that matured very early (29 and 32 days, respectively) and of a male that matured very late (90 days) did not change these results (data not shown). The limited sample size (*n* = 56) prevented us from fitting additional predictors, but a visual inspection of the data suggested that age at maturity was independent of maternal size (*r* = −.04, *t*_54_ = −0.30, *p* = .77; Figure 7e) and size at maturity (*r* = .18, *t*_54_ = 1.38, *p* = .17; Figure 7f).

## 4 DISCUSSION

Our results suggest that different per‐capita food levels are one of the selective agents that drive the evolution of divergent life histories in two populations of the Least Killifish. The most direct evidence was the stark difference in survival between TP and WR fish at the lower food level. There was a 75% mortality rate for TP fish at low food, compared with a 15% mortality rate for WR fish. At the higher food level, mortality rates were more similar between populations, 15% for TP fish and 0% for WR fish. The interpretation that WR fish are better able to survive under food limitation than TP fish is bolstered by the contributions of the individual cells to the chi‐square score, with the number dead in TP at low food being the major component. As a potential agent of selection, low per‐capita food availability primarily affected the earliest age classes, seeing as 62% of nonsurviving fish died before the age of 14 days, and 90% before the age of 28 days.

Our results offer tentative suggestions as to the mechanisms behind the differential survival of food‐limited TP and WR fish. It seems to us that the survival advantage of WR fish may have resulted from (at least) two factors: an increased efficiency of food use and a larger size at birth. An increased digestive efficiency, rather than a faster feeding rate, is suggested by the fact that all low‐food fish ingested the same amounts of food. They were housed individually, fed identical food rations, and consumed them completely shortly after receiving them, all pointing toward WR fish using the scarce resources available to them more efficiently. This does not mean that WR fish could not, additionally, have a faster feeding rate; this will need to be established in future studies quantifying between‐population variation in feeding rates.

A second survival advantage of WR fish when faced with food scarcity appears to be their larger size at birth. While the size difference between WR and TP offspring is not a new finding (see references in Table 1), the positive association between size at birth and survival to maturity is. Interestingly, the association was significant only in a statistical model from which population identity had been dropped as a predictor. When both population and size at birth were included as predictors, only the effect of population was significant. This suggests that the increased survival of WR fish is indeed associated with their larger average size at birth, but that, within populations, survival and size at birth were either uncorrelated, or correlated differently in WR and TP fish. Unfortunately, the shortage of fish from WR that died in the experiment (*n* = 3) prevented us from investigating these options statistically. However, it is worth noting that the three dead WR fish were of similar, if not slightly larger, size at birth than most surviving WR fish, while the 18 dead TP fish tended to be born smaller than survivors from TP (Figure 4b). Hence, the relationship between size at birth and survival may potentially differ between populations. In fish from TP, survival under low‐food conditions appears to be linked to being born relatively large, whereas other factors (perhaps increased food use efficiency) appear to be critical in fish from WR. Again, more research is needed on this count.

Integrating our new findings with previous knowledge shows that the TP and WR populations of Least Killifish display many characteristics of classical *r*‐ and *K*‐selection (Macarthur & Wilson, 1967; Pianka, 1970). TP is an unstable habitat, with large annual fluctuations in temperature (Leips & Travis, 1999) and a high risk of death from predation (references in Table 1). Food is most likely abundant, as primary productivity is high (Figure 2) and population density low (Table 1), with population size kept far below carrying capacity by predators. By contrast, in WR temperature is more constant, population density much higher, and the main cause of death likely starvation, as the combination of high density and low primary productivity creates intense intraspecific competition for food. As expected from theory (Macarthur & Wilson, 1967; Pianka, 1970), the *r*‐selected TP fish are smaller at maturity and give birth to more, but smaller, offspring than the *K*‐selected WR fish (Table 1). Presumably, the production of many small offspring, rather than fewer large ones, pays off in TP fish because the higher primary productivity of their habitat allows small fish to survive, while the larger number of offspring ensures that at least some offspring survive to maturity despite the high predation risk. While the present study merely reconfirmed some of these results, it adds two crucial details to the picture. First, TP fish had reduced survival to maturity, even at the higher food level, in agreement with investment into productivity at the cost of somatic maintenance and long‐term survival. Second, they were substantially more sensitive to food scarcity than WR fish, as expected from a population regulated by density‐independent processes.

We acknowledge that ours is a case study, and it is important to note its limitations. With only two populations studied, we cannot yet conclude that the higher ability of WR fish to tolerate poor food conditions is an adaptive evolutionary response to higher density and potential food scarcity. Instead, their low resource requirements could be due to nonadaptive reasons such as a founder effect. To prove that a greater ability to cope with low per‐capita food availability results from selection at higher density, more populations of *H. formosa* need to be studied. Only when the sensitivity to food limitation is generally found to be higher in highlow‐ than in high‐density populations, can the cause–effect relationship be established.

Neither do our results preclude other agents of selection from operating in WR and TP simultaneously. Social stress is important in TP fish, where the effects of social density on reproductive rate were stronger than the effects of food level (Leatherbury & Travis, 2019). We do not know if WR fish are less sensitive to this source of stress. However, demonstrating that the sensitivity to food limitation differs between WR and TP fish is a step forward in moving the study of density‐dependent selection toward a more biological and less phenomenological focus (cf. Engen et al., 2020).

A second aim of our study was to test whether a larger size at birth was associated with higher survival (which, across populations, it was; see above), a larger size at maturity, and earlier maturation. In our experiment, focal individuals were the second‐generation laboratory‐born offspring of mothers fed to satiation. A maternal environment effect on size at birth, occasionally found in *H. formosa* with respect to maternal food levels (Leatherbury & Travis, 2019; but see Travis et al., 1987), is thus likely small, with variation in size at birth primarily arising from additive genetic and maternal genetic effects (Kruuk & Hadfield, 2007). In agreement with this, we found that size at birth was significantly affected by maternal identity.

The influence of size at birth on body size later in life diminished gradually during the juvenile phase and was no longer detectable by the time that fish matured, causing size at birth and size at maturity to be uncorrelated (Figure 6c). This is similar to patterns in other studies of fish species in which maternal effects were important in early life but became less so with offspring age (Heath & Blouw, 1998; Venney et al., 2020). The result was in contrast to the longer‐lasting effects of the differential sizes at birth between WR and TP fish. The smaller initial size of TP offspring carried through to a smaller size at maturity, even though the actual somatic growth rates of immature fish did not differ between populations. This result illustrates the importance of distinguishing variation in maternal effects within populations from those that might be observed between populations.

Although, across populations, size at birth did not predict size at maturity, it affected the age when fish reached maturity. It did so in a very particular, food‐dependent manner. At low food levels, individuals that were larger at birth matured earlier. This was a substantial effect: an individual that was smaller than 6.5 mm at birth matured ~16 days later than one that was larger than 7.0 mm at birth (see Figure 7d). At high food levels, this relationship was weaker (~10 days). In other words, a larger size at birth enabled fish to mature relatively early even when they were kept at the lower food level. This result reinforces the conclusion drawn by Leips et al. (2013), who found that size at birth was inversely correlated with age at maturity, especially when fish were raised in competitive conditions. It also ties in with the hypothesis that the larger size at birth of WR fish is an adaptation to lower per‐capita resource availability.

Finally, this study extends our knowledge about life‐history differences between the sexes. The older age at maturation and thus larger size of males compared to females found here is consistent with an earlier study (Hale & Travis, 2015). Field data using otolith ring counts as indicators of age also suggest that females mature before males (J. Travis, unpublished data). We now additionally showed that size at birth and juvenile growth were identical in males and females, suggesting that the larger size at maturity of males results entirely from sex‐specific threshold rules for maturation, rather than from sex differences in initial sizes or growth rates.

## CONFLICT OF INTEREST

The authors have declared that no conflicts of interest exist.

## AUTHOR CONTRIBUTIONS

**Anja Felmy:** Data curation (equal); Formal analysis (lead); Funding acquisition (equal); Methodology (lead); Software (lead); Validation (equal); Visualization (lead); Writing‐original draft (equal); Writing‐review & editing (equal). **Jeff Leips:** Conceptualization (equal); Investigation (equal); Resources (equal); Validation (equal); Writing‐review & editing (supporting). **Joseph Travis:** Conceptualization (equal); Data curation (equal); Formal analysis (supporting); Funding acquisition (equal); Investigation (equal); Methodology (supporting); Project administration (lead); Resources (equal); Software (supporting); Supervision (lead); Validation (equal); Visualization (supporting); Writing‐original draft (equal); Writing‐review & editing (equal).

### OPEN RESEARCH BADGES

This article has earned an Open Data Badge for making publicly available the digitally‐shareable data necessary to reproduce the reported results. The complete dataset will be available at the Dryad Digital Repository: https://doi.org/10.5061/dryad.gtht76hm0. The entire code and model output, prepared using R Markdown, can be found in the Appendix S1.

## Supporting information

Appendix S1

## Data Availability

The complete dataset is available at the Dryad Digital Repository: https://doi.org/10.5061/dryad.gtht76hm0.

## References

[ece37490-bib-0001] Allaire, J. , Xie, Y. , McPherson, J. , Luraschi, J. , Ushey, K. , Atkins, A. , Wickham, H. , Cheng, J. , Chang, W. , & Iannone, R. (2020). rmarkdown: Dynamic Documents for R, version R package version 2.3. https://github.com/rstudio/rmarkdown

[ece37490-bib-0002] Aresco, M. J. , Travis, J. , & MacRae, P. S. D. (2015). Trophic interactions of turtles in a North Florida lake food web: Prevalence of omnivory. Copeia, 103, 343–356. 10.1643/CE-13-130

[ece37490-bib-0003] Auer, S. K. , Dick, C. A. , Metcalfe, N. B. , & Reznick, D. N. (2018). Metabolic rate evolves rapidly and in parallel with the pace of life history. Nature Communications, 9, 6. 10.1038/s41467-017-02514-z PMC5750215 29295982

[ece37490-bib-0004] Bassar, R. D. , Bryan, B. L. , Marshall, M. C. , Pringle, C. M. , Reznick, D. N. , & Travis, J. (2017). Local adaptation of fish consumers alters primary production through changes in algal community composition and diversity. Oikos, 126, 594–603. 10.1111/oik.03965

[ece37490-bib-0005] Berec, L. , Kramer, A. M. , Bernhauerova, V. , & Drake, J. M. (2018). Density‐dependent selection on mate search and evolution of Allee effects. Journal of Animal Ecology, 87, 24–35. 10.1111/1365-2656.12662 28240356

[ece37490-bib-0006] Boyce, M. S. (1984). Restitution of r‐selection and K‐selection as a model of density‐dependent natural selection. Annual Review of Ecology and Systematics, 15, 427–447.

[ece37490-bib-0007] Brooks, M. E. , Kristensen, K. , Benthem, K. J. , Magnusson, A. , Berg, C. W. , Nielsen, A. , Skaug, H. J. , Mächler, M. , & Bolker, B. M. (2017). glmmTMB balances speed and flexibility among packages for zero‐inflated generalized linear mixed modeling. The R Journal, 9, 378–400. 10.32614/RJ-2017-066

[ece37490-bib-0008] Bull, J. J. , Millstein, J. , Orcutt, J. , & Wichman, H. A. (2006). Evolutionary feedback mediated through population density, illustrated with viruses in chemostats. American Naturalist, 167, E39–E51. 10.1086/499374 16670974

[ece37490-bib-0009] Charlesworth, B. (1994). Evolution in age‐structured populations (2nd ed.). Cambridge University Press.

[ece37490-bib-0010] Eklund, A. (2016). beeswarm: the bee swarm plot, an alternative to stripchart, version R package version 0.2.3. https://CRAN.R‐project.org/package=beeswarm

[ece37490-bib-0011] El‐Sabaawi, R. W. , Marshall, M. C. , Bassar, R. D. , Lopez‐Sepulcre, A. , Palkovacs, E. P. , & Dalton, C. (2015). Assessing the effects of guppy life history evolution on nutrient recycling: From experiments to the field. Freshwater Biology, 60, 590–601. 10.1111/fwb.12507

[ece37490-bib-0012] El‐Sabaawi, R. W. , Zandonà, E. , Kohler, T. J. , Marshall, M. C. , Moslemi, J. M. , Travis, J. , López‐Sepulcre, A. , Ferriére, R. , Pringle, C. M. , Thomas, S. A. , Reznick, D. N. , & Flecker, A. S. (2012). Widespread intraspecific organismal stoichiometry among populations of the Trinidadian guppy. Functional Ecology, 26, 666–676. 10.1111/j.1365-2435.2012.01974.x

[ece37490-bib-0013] Engen, S. , & Saether, B. E. (2016). Optimal age of maturity in fluctuating environments under r‐ and K‐selection. Oikos, 125, 1577–1585.

[ece37490-bib-0014] Engen, S. , & Saether, B. E. (2017). r‐ and K‐selection in fluctuating populations is determined by the evolutionary trade‐off between two fitness measures: Growth rate and lifetime reproductive success. Evolution, 71, 167–173. 27804129 10.1111/evo.13104

[ece37490-bib-0015] Engen, S. , Wright, J. , Araya‐Ajoy, Y. G. , & Saether, B. E. (2020). Phenotypic evolution in stochastic environments: The contribution of frequency‐ and density‐dependent selection. Evolution, 74, 1923–1941. 10.1111/evo.14058 32656772

[ece37490-bib-0016] Gutierrez, Y. , Fresch, M. , Ott, D. , Brockmeyer, J. , & Scherber, C. (2020). Diet composition and social environment determine food consumption, phenotype and fecundity in an omnivorous insect. Royal Society Open Science, 7, 16. 10.1098/rsos.200100 PMC7211883 32431901

[ece37490-bib-0017] Hale, R. E. , & Travis, J. (2015). Effects of water chemistry on the life history of the Least Killifish *Heterandria* *formosa* and the absence of evidence for local adaptation. Copeia, 103, 51–57.

[ece37490-bib-0018] Heath, D. D. , & Blouw, D. M. (1998). Are maternal effects in fish adaptive or merely physiological side effects? In T. A. Mousseau , & C. W. Fox (Eds.), Maternal effects as adaptations (pp. 178–201). Oxford University Press.

[ece37490-bib-0019] Joshi, A. , Knight, C. D. , & Mueller, L. D. (1996). Genetics of larval urea tolerance in *Drosophila melanogaster* . Heredity, 77, 33–39. 10.1038/hdy.1996.105 8682692

[ece37490-bib-0020] Joshi, A. , & Mueller, L. D. (1988). Evolution of higher feeding rate in *Drosophila* due to density‐dependent natural selection. Evolution, 42, 1090–1093. 28581181 10.1111/j.1558-5646.1988.tb02527.x

[ece37490-bib-0021] Joshi, A. , & Mueller, L. D. (1993). Directional and stabilizing density‐dependent natural selection for pupation height in *Drosophila melanogaster* . Evolution, 47, 176–184. 28568099 10.1111/j.1558-5646.1993.tb01208.x

[ece37490-bib-0022] Joshi, A. , & Mueller, L. D. (1996). Density‐dependent natural selection in *Drosophila*: Trade‐offs between larval food acquisition and utilization. Evolutionary Ecology, 10, 463–474. 10.1007/BF01237879

[ece37490-bib-0023] Joshi, A. , Wu, W. P. , & Mueller, L. D. (1998). Density‐dependent natural selection in *Drosophila*: Adaptation to adult crowding. Evolutionary Ecology, 12, 363–376. 10.1023/A:1006508418493

[ece37490-bib-0024] Kruuk, L. E. B. , & Hadfield, J. D. (2007). How to separate genetic and environmental causes of similarity between relatives. Journal of Evolutionary Biology, 20, 1890–1903. 10.1111/j.1420-9101.2007.01377.x 17714306

[ece37490-bib-0025] Landy, J. A. , & Travis, J. (2015). Shape variation in the least killifish: Ecological associations of phenotypic variation and the effects of a common garden. Ecology and Evolution, 5, 5616–5631. 10.1002/ece3.1780 27069611 PMC4813119

[ece37490-bib-0026] Landy, J. A. , & Travis, J. (2018). Unique maternal and environmental effects on the body morphology of the Least Killifish, *Heterandria* *formosa* . Ecology and Evolution, 8, 6267–6279. 10.1002/ece3.4166 PMC6024122 29988417

[ece37490-bib-0027] Leatherbury, K. N. , & Travis, J. (2019). The effects of food level and social density on reproduction in the Least Killifish, *Heterandria* *formosa* . Ecology and Evolution, 9, 100–110. 30680099 10.1002/ece3.4634 PMC6341976

[ece37490-bib-0028] Leips, J. , Richardson, J. M. L. , Rodd, F. H. , & Travis, J. (2009). Adaptive maternal adjustments of offspring size in response to conspecific density in two populations of the Least Killifish, *Heterandria* *formosa* . Evolution, 63, 1341–1347. 19425199 10.1111/j.1558-5646.2009.00631.x

[ece37490-bib-0029] Leips, J. , Rodd, F. H. , & Travis, J. (2013). The adaptive significance of population differentiation in offspring size of the Least Killifish, *Heterandria* *formosa* . Ecology and Evolution, 3, 948–960. 23610636 10.1002/ece3.509 PMC3631406

[ece37490-bib-0030] Leips, J. , & Travis, J. (1999). The comparative expression of life‐history traits and its relationship to the numerical dynamics of four populations of the Least Killifish. Journal of Animal Ecology, 68, 595–616. 10.1046/j.1365-2656.1999.00311.x

[ece37490-bib-0031] Leips, J. , Travis, J. , & Rodd, F. H. (2000). Genetic influences on experimental population dynamics of the Least Killifish. Ecological Monographs, 70, 289–309.

[ece37490-bib-0032] Macarthur, R. H. , & Wilson, E. O. (1967). The theory of island biogeography. Princeton University Press.

[ece37490-bib-0033] MacRae, P. S. D. , & Travis, J. (2014). The contribution of abiotic and biotic factors to spatial and temporal variation in population density of the Least Killifish, *Heterandria* *formosa* . Environmental Biology of Fishes, 97, 1–12. 10.1007/s10641-013-0117-7

[ece37490-bib-0034] Mueller, L. D. (1988). Density‐dependent population‐growth and natural selection in food‐limited environments – The *Drosophila* model. American Naturalist, 132, 786–809. 10.1086/284890

[ece37490-bib-0035] Mueller, L. D. (1990). Density‐dependent natural selection does not increase efficiency. Evolutionary Ecology, 4, 290–297. 10.1007/BF02270928

[ece37490-bib-0036] Mueller, L. D. (1997). Theoretical and empirical examination of density‐dependent selection. Annual Review of Ecology and Systematics, 28, 269–288. 10.1146/annurev.ecolsys.28.1.269

[ece37490-bib-0037] Mueller, L. D. , Joshi, A. , & Borash, D. J. (2000). Does population stability evolve? Ecology, 81, 1273–1285.

[ece37490-bib-0038] Pianka, E. R. (1970). On r‐ and K‐selection. The American Naturalist, 104, 592–597. 10.1086/282697

[ece37490-bib-0039] R Core Team (2020). R: A language and environment for statistical computing. R Foundation for Statistical Computing. https://www.R‐project.org/

[ece37490-bib-0040] Richardson, J. M. L. , Gunzburger, M. S. , & Travis, J. (2006). Variation in predation pressure as a mechanism underlying differences in numerical abundance between populations of the poeciliid fish *Heterandria* *formosa* . Oecologia, 147, 596–605. 10.1007/s00442-005-0306-y 16341890

[ece37490-bib-0041] Sarangi, M. , Nagarajan, A. , Dey, S. , Bose, J. , & Joshi, A. (2016). Evolution of increased larval competitive ability in *Drosophila melanogaster* without increased larval feeding rate. Journal of Genetics, 95, 491–503. 10.1007/s12041-016-0656-8 27659320

[ece37490-bib-0042] Schrader, M. , & Travis, J. (2005). Population differences in pre‐ and post‐fertilization offspring provisioning in the Least Killifish, *Heterandria* *formosa* . Copeia, 2005(3), 649–656.

[ece37490-bib-0043] Schrader, M. , & Travis, J. (2008). Testing the viviparity‐driven‐conflict hypothesis: Parent‐offspring conflict and the evolution of reproductive isolation in a poeciliid fish. The American Naturalist, 172, 806–817. 10.1086/592999 18950276

[ece37490-bib-0044] Schrader, M. , & Travis, J. (2012). Assessing the roles of population density and predation risk in the evolution of offspring size in populations of a placental fish. Ecology and Evolution, 2, 1480–1490. 10.1002/ece3.255 22957156 PMC3434941

[ece37490-bib-0045] Than, A. T. , Ponton, F. , & Morimoto, J. (2020). Integrative developmental ecology: A review of density‐dependent effects on life‐history traits and host‐microbe interactions in non‐social holometabolous insects. Evolutionary Ecology, 34, 659–680. 10.1007/s10682-020-10073-x

[ece37490-bib-0046] Travis, J. , Farr, J. A. , Henrich, S. , & Cheong, R. T. (1987). Testing theories of clutch overlap with the reproductive ecology of *Heterandria* *formosa* . Ecology, 68, 611–623. 10.2307/1938466

[ece37490-bib-0047] Travis, J. , & Trexler, J. C. (1986). Interactions among factors affecting growth, development and survival in experimental populations of *Bufo* *terrestris* (Anura, Bufonidae). Oecologia, 69, 110–116. 10.1007/BF00399045 28311692

[ece37490-bib-0048] Tung, S. , Rajamani, M. , Joshi, A. , & Dey, S. (2019). Complex interaction of resource availability, life‐history and demography determines the dynamics and stability of stage‐structured populations. Journal of Theoretical Biology, 460, 1–12. 10.1016/j.jtbi.2018.10.019 30300650

[ece37490-bib-0049] Venney, C. J. , Love, O. P. , Drown, E. J. , & Heath, D. D. (2020). DNA methylation profiles suggest intergenerational transfer of maternal effects. Molecular Biology and Evolution, 37, 540–548. 10.1093/molbev/msz244 31651942

[ece37490-bib-0050] Walsh, M. R. , & Reznick, D. N. (2010). Influence of the indirect effects of guppies on life‐history evolution in *Rivulus* *hartii* . Evolution, 64, 1583–1593. 10.1111/j.1558-5646.2009.00922.x 20015237

[ece37490-bib-0051] Warner, S. C. , Dunson, W. A. , & Travis, J. (1991). Interaction of pH, density, and priority effects on the survivorship and growth of two species of hylid tadpoles. Oecologia, 88, 331–339. 10.1007/BF00317575 28313793

[ece37490-bib-0052] Wilbur, H. M. (1977). Interactions of food level and population density in *Rana* *sylvatica* . Ecology, 58, 206–209. 10.2307/1935124

[ece37490-bib-0053] Wright, J. , Bolstad, G. H. , Araya‐Ajoy, Y. G. , & Dingemanse, N. J. (2019). Life‐history evolution under fluctuating density‐dependent selection and the adaptive alignment of pace‐of‐life syndromes. Biological Reviews, 94, 230–247. 10.1111/brv.12451 30019372

[ece37490-bib-0054] Xie, Y. , Allaire, J. , & Grolemund, G. (2018). R Markdown: The definitive guide. Chapman and Hall/CRC.

[ece37490-bib-0055] Zandonà, E. , Auer, S. K. , Kilham, S. S. , Howard, J. L. , López‐Sepulcre, A. , O’Connor, M. P. , Bassar, R. D. , Osorio, A. , Pringle, C. M. , & Reznick, D. N. (2011). Diet quality and prey selectivity correlate with life histories and predation regime in Trinidadian guppies. Functional Ecology, 25, 964–973. 10.1111/j.1365-2435.2011.01865.x

[ece37490-bib-0056] Zar, J. H. (1996). Sampling a binomial population. In S. Fisher , & S. L. Snavely (Eds.), Biostatistical analysis (p. 523). Prentice‐Hall Inc.

