# Supplementary material for: Ancestral ecological regime shapes reaction to food limitation in the Least Killifish, Heterandria formosa
Source: Ecol Evol. 2021 Apr 6;11(11):6391–405. doi: 10.1002/ece3.7490 (PMC8207351; doi:10.1002/ece3.7490)

Supporting Information for

Ancestral ecological regime shapes reaction to food limitation in the Least Killifish, *Heterandria formosa*

Anja Felmy, Jeff Leips, Joseph Travis

R Markdown Document

12 03 2021

Table of Contents

1. Preparations ... 3
1.1 Import data and choose data type ... 3
1.2 Load required packages ... 3
1.3 Set values to NA for individual H7 with potential measurement errors ... 4
1.4 Correlation between photographic length measurements and dry weights ... 4
2. Water quality data ... 4
2.1 Difference between sampling localities ... 4
2.2 Figure 2 ... 5
3. Analysis of size at birth ... 6
3.1 Distribution of data ... 6
3.2 Model 1: size at birth ... 7
3.3 Model 2: size at birth (test of effect of sex) ... 9
3.4 Model 3: size at birth (test of influence of outliers) ... 11
3.5 Model 4: size at birth (test of effect of sex when excluding outliers) ... 12
3.6 Figure 3 ... 14
4. Analysis of survival to sexual maturity ... 15
4.1 At what age did fish die? ... 15
4.2 Identical effects of population of origin and food levels ... 16
4.3 Model 5: survival to sexual maturity ... 17
4.4 Model 6: survival to sexual maturity (test effect of size at birth once population is removed as a predictor) ... 18
4.5 Chi-square test for survival to sexual maturity ... 19
4.6 Computing binomial standard errors for survival rates ... 20
4.7 Figure 4 ... 20

5. Analysis of size at 14 days 22
5.1 Distribution of size at 14 days 22
5.2 Computing proportional differences between populations and food levels 23
5.3 Model 7: size at 14 days 23
5.4 Model 8: size at 14 days (test of influence of outliers) 25
5.5 Figure 5 26
6. Analysis of size as a juvenile and at maturity 28
6.1 Data preparation 28
6.2 Distribution of size as a juvenile and at maturity 29
6.3 Model 9: size as a juvenile and at maturity 30
6.4 Preparations for figure 6 31
6.5 Figure 6 33
7. Analysis of age at sexual maturity 35
7.1 Distribution of age at sexual maturity 35
7.2 Computing proportional differences between populations, food levels and sexes 36
7.3 Model 10: age at sexual maturity 37
7.4 Model 11: age at sexual maturity (test of influence of outliers) 38
7.5 Correlation between age at maturity and other variables 40
7.6 Difference in age at maturity between fish born large or small as a function of food level 41
7.7 Figure 7 42

1. Preparations

1.1 Import data and choose data type

```
# Fish data
d<-read.csv("Fish data.csv", header=T, sep=";")

d$FISHID  <-as.factor(d$FISHID)   # individual identity
d$MOM     <-as.factor(d$MOM)      # maternal identity
d$LOCATION<-as.factor(d$LOCATION) # population of origin
d$FOOD    <-as.factor(d$FOOD)     # experimental food level
d$SEX     <-as.factor(d$SEX)      # sex

d$MOMSZ <-as.numeric(d$MOMSZ)  # maternal size [mm]
d$ISIZE <-as.numeric(d$ISIZE)  # size at birth / initial size [mm]
d$SMAT  <-as.numeric(d$SMAT)   # size at sexual maturity [mm]
d$AGEMAT<-as.numeric(d$AGEMAT) # age at sexual maturity [days]
d$SZ14  <-as.numeric(d$SZ14)   # size at 14 days [mm]
d$SZ28  <-as.numeric(d$SZ28)   # size at 28 days [mm]
d$SZ42  <-as.numeric(d$SZ42)   # size at 42 days [mm]
d$SURV  <-as.factor(d$SURV)    # survival to sexual maturity [0=dead, 1=alive
]

d$locfood   <-as.factor(d$locfood)    # population-food level combination
d$locsex    <-as.factor(d$locsex)     # population-sex combination
d$locfoodsex<-as.factor(d$locfoodsex) # population-food level-sex combination
d$foodsex   <-as.factor(d$foodsex)    # food level-sex combination
d$survloc   <-as.factor(d$survloc)    # survival-population combination

# Water data
w<-read.csv("Water data.csv", header=T, sep=";")

w$Location<-as.factor(w$Location)
w$Year    <-as.factor(w$Year)

# Measuring trial
m<-read.csv("Measuring trial.csv", header=T, sep=";")

m$FISHID<-as.factor(m$FISHID)
```

1.2 Load required packages

```
library(glmmTMB)
library(beeswarm)
library(dae)
```

1.3 Set values to NA for individual H7 with potential measurement errors

```
# set sizes and age at maturity of H7 to NA
d$ISIZE [d$FISHID %in% "H7"]<-NA
d$SZ14  [d$FISHID %in% "H7"]<-NA
d$SZ28  [d$FISHID %in% "H7"]<-NA
d$SZ42  [d$FISHID %in% "H7"]<-NA
d$SMAT  [d$FISHID %in% "H7"]<-NA
d$AGEMAT[d$FISHID %in% "H7"]<-NA
```

1.4 Correlation between photographic length measurements and dry weights

```
cor.test(m$SL, m$MASS)

##
##  Pearson's product-moment correlation
##
## data:  m$SL and m$MASS
## t = 5.0148, df = 18, p-value = 8.992e-05
## alternative hypothesis: true correlation is not equal to 0
## 95 percent confidence interval:
##  0.4846446 0.9014229
## sample estimates:
##       cor
## 0.7634351
```

2. Water quality data

2.1 Difference between sampling localities

```
w$TotalN[w$Location %in% "Trout"]/w$TotalN[w$Location %in% "Wacissa"]

## [1] 1.0683112 0.7734488 1.1933333

w$OrganicN[w$Location %in% "Trout"]/w$OrganicN[w$Location %in% "Wacissa"]

## [1] 2.502222 2.447489 3.781690

w$ChlorophyllA[w$Location %in% "Trout"]/w$ChlorophyllA[w$Location %in% "Wacis
sa"]

## [1] 14.619048  5.218182  8.120000

w$TotalN/w$OrganicN

## [1] 1.000000 1.000000 1.000000 2.342222 3.164384 3.169014
```

2.2 Figure 2

```
# data preparation
w10<-w[w$Year %in% "2010",]
w11<-w[w$Year %in% "2011",]
w13<-w[w$Year %in% "2013",]

TotN10<-w10$TotalN
OrgN10<-w10$OrganicN
Chlo10<-w10$ChlorophyllA

TotN11<-w11$TotalN
OrgN11<-w11$OrganicN
Chlo11<-w11$ChlorophyllA

TotN13<-w13$TotalN
OrgN13<-w13$OrganicN
Chlo13<-w13$ChlorophyllA

par(mfrow=c(1,3))
par(mar=c(4.5,5.2,2,1), xpd=T, cex=0.66)

# Figure 2a
barplot(height=cbind(a = Chlo10,
                     b = Chlo11,
                     c = Chlo13),
           beside=TRUE,
             col=c("green1","lightskyblue"),
             xlab="", yaxt="n", cex.axis=1.6, cex.lab=1.6,cex.names=1.6, las=
1,
             names.arg=c("2010", "2011", "2013"),
               space=c(0, 0.5), ylim=c(0,3.25))

title(ylab="Chlorophyll a [mg/cubic metre]", mgp=c(3.5, 1, 2.0), cex.lab=1.6)
axis(2, at=c(0, 0.5, 1.0, 1.5, 2.0, 2.5, 3.0), cex.axis=1.6, las=1)
title(xlab="Sampling year", mgp=c(3, 1.5, 0), cex.lab=1.6)
legend("topright", legend=c("Trout P.", "Wacissa R."), fill=c("green1", "ligh
tskyblue"),
col=c("green1", "lightskyblue"), bty="n", cex=1.6, inset=c(0.05, -0.12))
text(-1.8, 3.35, "a", font=2, cex=2)

# Figure 2b
barplot(height=cbind(a = OrgN10,
                     b = OrgN11,
                     c = OrgN13),
           beside=TRUE,
             col=c("green1","lightskyblue"),
             xlab="", yaxt="n", cex.axis=1.6, cex.lab=1.6,cex.names=1.6, las=
1,
             names.arg=c("2010", "2011", "2013"),
```

```
                space=c(0, 0.5), ylim=c(0,0.77))

title(ylab="Organic nitrogen [mg/litre]", mgp=c(4, 1, 2.0), cex.lab=1.6)
axis(2, at=c(0, 0.25, 0.5, 0.75), cex.axis=1.6, las=1)
title(xlab="Sampling year", mgp=c(3, 1.5, 0), cex.lab=1.6)
text(-1.8, 0.8, "b", font=2, cex=2)

# Figure 2c
barplot(height=cbind(a = TotN10,
                     b = TotN11,
                     c = TotN13),
           beside=TRUE,
             col=c("green1","lightskyblue"),
             xlab="", yaxt="n", cex.axis=1.6, cex.lab=1.6,cex.names=1.6, las=
1,
             names.arg=c("2010", "2011", "2013"),
               space=c(0, 0.5), ylim=c(0,0.77))

title(ylab="Total nitrogen [mg/litre]", mgp=c(4, 1, 2.0), cex.lab=1.6)
axis(2, at=c(0, 0.25, 0.5, 0.75), cex.axis=1.6, las=1)
title(xlab="Sampling year", mgp=c(3, 1.5, 0), cex.lab=1.6)
text(-1.8, 0.79, "c", font=2, cex=2)
```

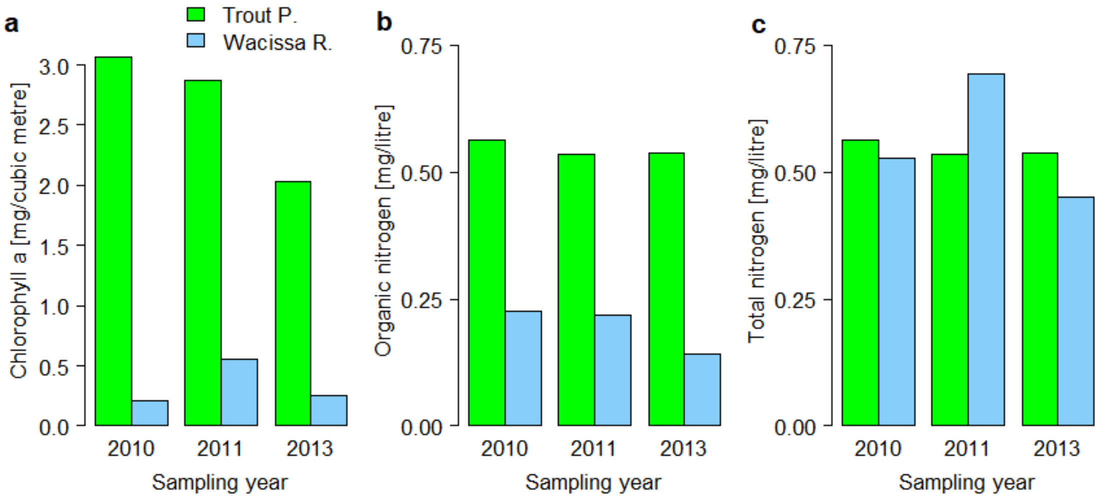

3. Analysis of size at birth

3.1 Distribution of data

```
# two-sided Kolmogorov-Smirnov test, showing no significant deviation from no
rmality
ks.test(d$ISIZE, pnorm, mean(d$ISIZE, na.rm=T), sd(d$ISIZE, na.rm=T))
##  One-sample Kolmogorov-Smirnov test
##
## data:  d$ISIZE
```

```
## D = 0.079534, p-value = 0.6997
## alternative hypothesis: two-sided

# histogram and quantile-quantile plot, also indicating that data are approxi
mately normally distributed
par(mfrow=c(1,2))
hist(d$ISIZE, breaks=50, main="", xlab="Size at birth [mm]", las=1)
qqnorm(d$ISIZE, pch = 1, frame = FALSE, main="", las=1)
qqline(d$ISIZE, col = "steelblue", lwd = 2)
```

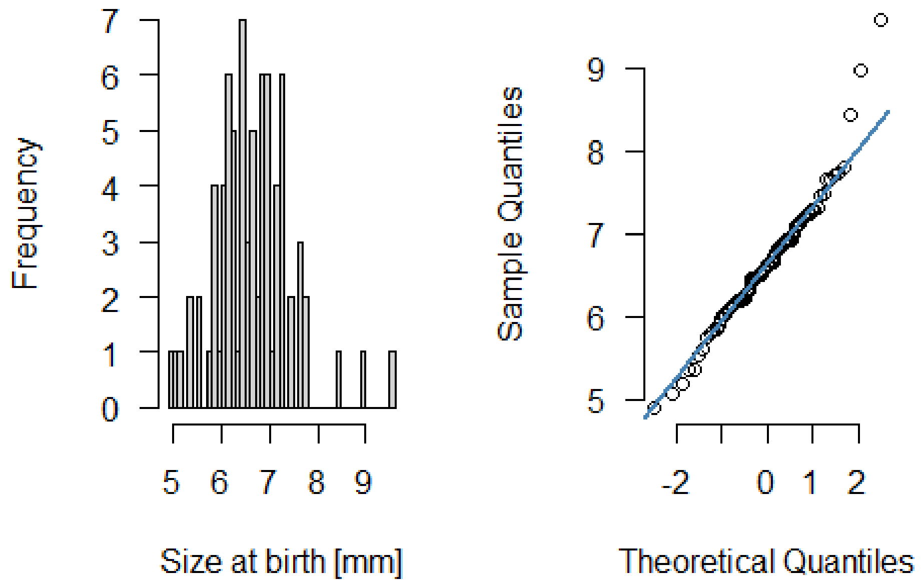

3.2 Model 1: size at birth

```
nrow(d[is.na(d$ISIZE)==F,]) # sample size: 79 individuals

## [1] 79

# GLMM with Gaussian errors including the population of origin, experimental
food level, maternal size, and the interaction between population and materna
l size as fixed effects, and maternal identity as a random effect
m1<-glmmTMB(ISIZE ~ LOCATION + FOOD + MOMSZ + LOCATION:MOMSZ + (1|MOM),
         family=gaussian, data=d)
summary(m1)

##  Family: gaussian  ( identity )
## Formula:          ISIZE ~ LOCATION + FOOD + MOMSZ + LOCATION:MOMSZ + (1 |
MOM)
## Data: d
##
```

```
##       AIC      BIC   logLik deviance df.resid
##     151.9    168.5    -68.9    137.9       72
##
## Random effects:
##
## Conditional model:
##  Groups   Name        Variance Std.Dev.
##  MOM      (Intercept) 0.1412   0.3757
##  Residual             0.2376   0.4875
## Number of obs: 79, groups:  MOM, 31
##
## Dispersion estimate for gaussian family (sigma^2): 0.238
##
## Conditional model:
##                       Estimate Std. Error z value Pr(>|z|)
## (Intercept)            4.32320    1.31649   3.284  0.00102 **
## LOCATIONWacissa        3.81366    1.65054   2.311  0.02086 *
## FOODLow               -0.30948    0.11337  -2.730  0.00633 **
## MOMSZ                  0.09054    0.05625   1.610  0.10746
## LOCATIONWacissa:MOMSZ -0.12943    0.07039  -1.839  0.06596 .
## ---
## Signif. codes:  0 '***' 0.001 '**' 0.01 '*' 0.05 '.' 0.1 ' ' 1

# diagnostic plot of residuals versus fitted values
res_m1<-resid(m1)
fitted_m1<-fitted(m1)
plot(res_m1~fitted_m1, las=1)
```

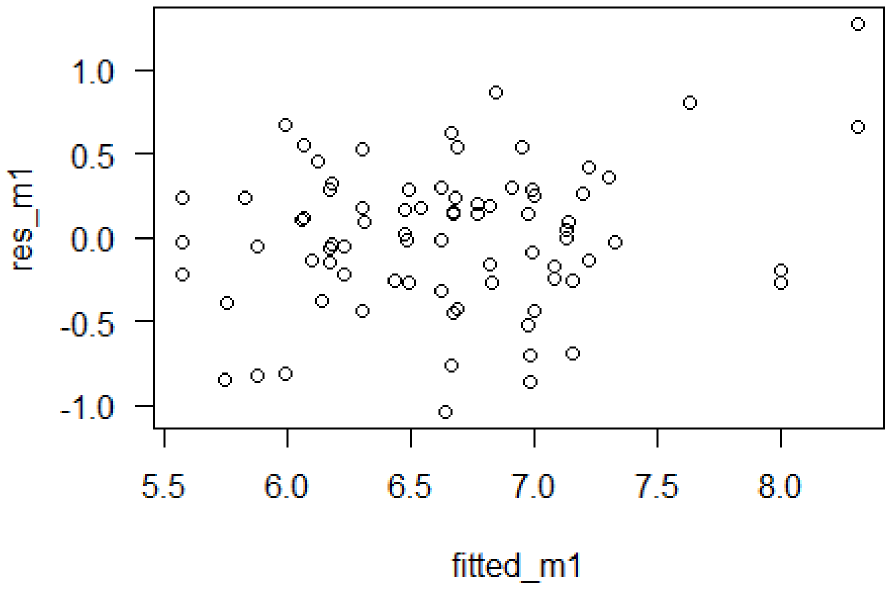

```
# obtaining a p-value for effect of maternal identity with a log-likelihood r
atio test comparing the full model to one without random effects
m1a<-glmmTMB(ISIZE ~ LOCATION + FOOD + MOMSZ + LOCATION:MOMSZ,
         family=gaussian, data=d)
anova(m1, m1a)
```

```
## Data: d
## Models:
## m1a: ISIZE ~ LOCATION + FOOD + MOMSZ + LOCATION:MOMSZ, zi=~0, disp=~1
## m1: ISIZE ~ LOCATION + FOOD + MOMSZ + LOCATION:MOMSZ + (1 | MOM), zi=~0, d
isp=~1
##     Df    AIC    BIC  logLik deviance  Chisq Chi Df Pr(>Chisq)
## m1a  6 159.32 173.53 -73.658   147.32
## m1   7 151.87 168.45 -68.934   137.87 9.4479      1   0.002114 **
## ---
## Signif. codes:  0 '***' 0.001 '**' 0.01 '*' 0.05 '.' 0.1 ' ' 1
```

3.3 Model 2: size at birth (test of effect of sex)

This model uses only fish with known sex, allowing us to test for a size difference between the sexes.

```
# making a new dataset that only contains fish with known sex
dsub1<-d[d$SEX %in% c("female", "male"),]
nrow(dsub1[is.na(dsub1$ISIZE)==F,]) # 58 individuals with known sex
```

```
## [1] 58
```

```
# GLMM using the subset of fish with known sex, including the same predictors
as model 1, plus sex
m2<-glmmTMB(ISIZE ~ LOCATION + FOOD + SEX + MOMSZ + LOCATION:MOMSZ + (1|MOM),
         family=gaussian, data=dsub1)
summary(m2)
```

```
##  Family: gaussian  ( identity )
## Formula:
## ISIZE ~ LOCATION + FOOD + SEX + MOMSZ + LOCATION:MOMSZ + (1 |      MOM)
## Data: dsub1
##
##      AIC      BIC   logLik deviance df.resid
##    111.5    128.0    -47.8     95.5       50
##
## Random effects:
##
## Conditional model:
##  Groups   Name        Variance Std.Dev.
##  MOM      (Intercept) 0.1700   0.4123
##  Residual             0.1879   0.4335
## Number of obs: 58, groups:  MOM, 27
##
```

```
## Dispersion estimate for gaussian family (sigma^2): 0.188
##
## Conditional model:
##                          Estimate Std. Error z value Pr(>|z|)
## (Intercept)               3.43965    1.60680   2.141 0.032300 *
## LOCATIONWacissa           4.96425    1.94202   2.556 0.010581 *
## FOODLow                  -0.45868    0.13073  -3.509 0.000451 ***
## SEXmale                  -0.26161    0.14172  -1.846 0.064889 .
## MOMSZ                     0.13530    0.06778   1.996 0.045915 *
## LOCATIONWacissa:MOMSZ -0.17768    0.08143  -2.182 0.029105 *
## ---
## Signif. codes:  0 '***' 0.001 '**' 0.01 '*' 0.05 '.' 0.1 ' ' 1

# diagnostic plot of residuals versus fitted values
res_m2<-resid(m2)
fitted_m2<-fitted(m2)
plot(res_m2~fitted_m2, las=1)
```

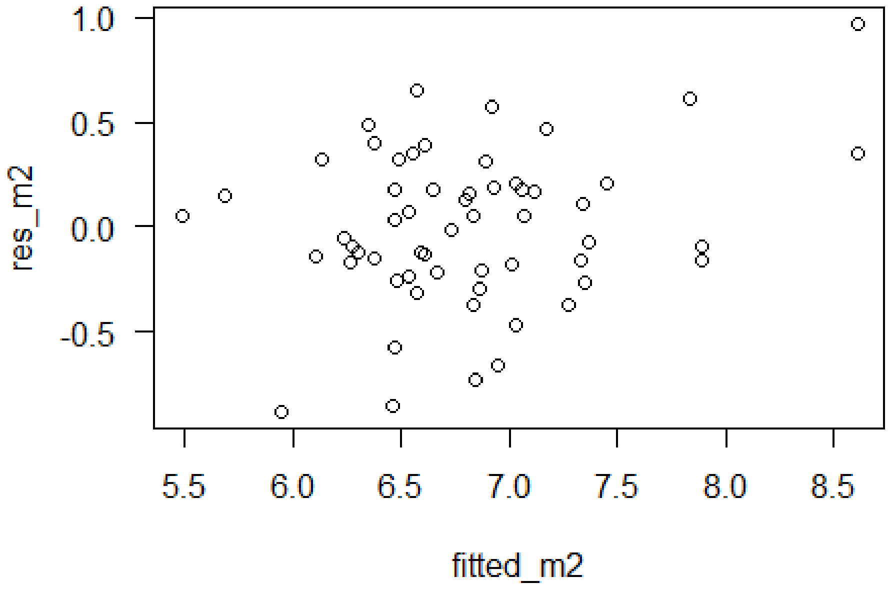


```
# obtaining a p-value for effect of maternal identity
m2a<-glmmTMB(ISIZE ~ LOCATION + FOOD + SEX + MOMSZ + LOCATION:MOMSZ,
         family=gaussian, data=dsub1)
anova(m2, m2a)

## Data: dsub1
## Models:
## m2a: ISIZE ~ LOCATION + FOOD + SEX + MOMSZ + LOCATION:MOMSZ, zi=~0, disp=~
1
## m2: ISIZE ~ LOCATION + FOOD + SEX + MOMSZ + LOCATION:MOMSZ + (1 | , zi=~0,
```

```
disp=~1
## m2:     MOM), zi=~0, disp=~1
##     Df    AIC    BIC  logLik deviance  Chisq Chi Df Pr(>Chisq)
## m2a  7 121.72 136.14 -53.860  107.720
## m2   8 111.53 128.01 -47.763   95.527 12.193      1  0.0004796 ***
## ---
## Signif. codes:  0 '***' 0.001 '**' 0.01 '*' 0.05 '.' 0.1 ' ' 1
```

3.4 Model 3: size at birth (test of influence of outliers)

This model excludes the three fish with very large sizes at birth, identified as potential outliers in the QQ-plot.

```
# making a new dataset that only contains fish with a size at birth of < 8mm
dsub2<-d[d$ISIZE < 8,]
nrow(dsub2[is.na(dsub2$ISIZE)==F,]) # 76 individuals included in reduced data
set

## [1] 76

# GLMM using the subset of fish with size at birth < 8mm, including the same
predictors as model 1
m3<-glmmTMB(ISIZE ~ LOCATION + FOOD + MOMSZ + LOCATION:MOMSZ + (1|MOM),
         family=gaussian, data=dsub2)
summary(m3)

##  Family: gaussian  ( identity )
## Formula:          ISIZE ~ LOCATION + FOOD + MOMSZ + LOCATION:MOMSZ + (1 |
MOM)
## Data: dsub2
##
##      AIC      BIC   logLik deviance df.resid
##    127.5    143.8    -56.7    113.5       69
##
## Random effects:
##
## Conditional model:
##  Groups   Name        Variance Std.Dev.
##  MOM      (Intercept) 0.05399  0.2323
##  Residual             0.21615  0.4649
## Number of obs: 76, groups:  MOM, 31
##
## Dispersion estimate for gaussian family (sigma^2): 0.216
##
## Conditional model:
##                         Estimate Std. Error z value Pr(>|z|)
## (Intercept)              4.11621    1.05789   3.891 9.99e-05 ***
## LOCATIONWacissa          2.94386    1.32053   2.229   0.0258 *
## FOODLow                 -0.16896    0.11073  -1.526   0.1270
## MOMSZ                    0.09583    0.04521   2.120   0.0340 *
```

```
## LOCATIONWacissa:MOMSZ -0.09706    0.05625  -1.726   0.0844 .
## ---
## Signif. codes:  0 '***' 0.001 '**' 0.01 '*' 0.05 '.' 0.1 ' ' 1
```

```
# diagnostic plot of residuals versus fitted values
res_m3<-resid(m3)
fitted_m3<-fitted(m3)
plot(res_m3~fitted_m3, las=1)
```

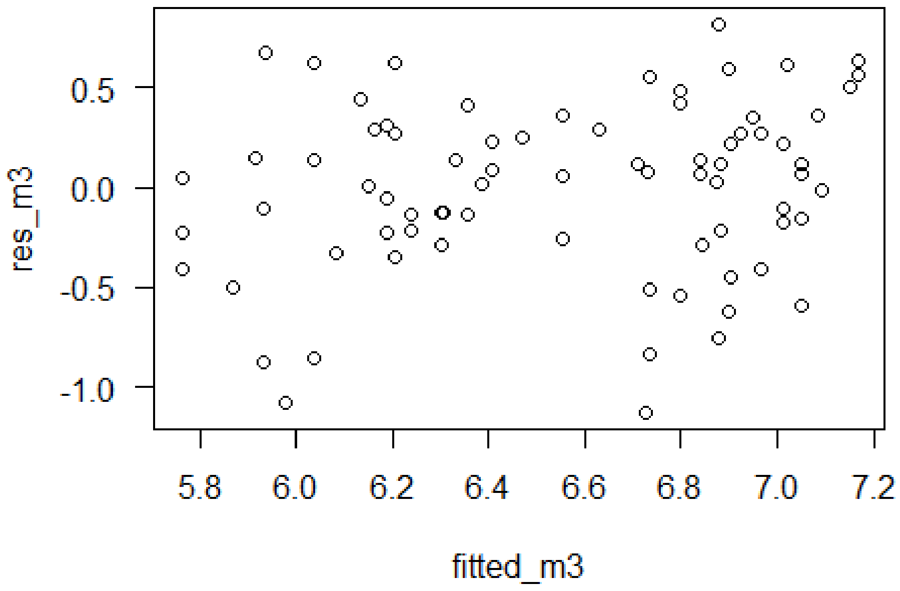


```
# obtaining a p-value for effect of maternal identity
m3a<-glmmTMB(ISIZE ~ LOCATION + FOOD + MOMSZ + LOCATION:MOMSZ,
         family=gaussian, data=dsub2)
anova(m3, m3a)
```

```
## Data: dsub2
## Models:
## m3a: ISIZE ~ LOCATION + FOOD + MOMSZ + LOCATION:MOMSZ, zi=~0, disp=~1
## m3: ISIZE ~ LOCATION + FOOD + MOMSZ + LOCATION:MOMSZ + (1 | MOM), zi=~0, d
isp=~1
##     Df    AIC    BIC  logLik deviance  Chisq Chi Df Pr(>Chisq)
## m3a  6 127.86 141.84 -57.928   115.86
## m3   7 127.50 143.81 -56.748   113.50 2.3601      1     0.1245
```

3.5 Model 4: size at birth (test of effect of sex when excluding outliers)

This model excludes the three fish with very large sizes at birth and fish with unknown sex.

```
# making a new dataset that only contains fish with a size at birth of < 8mm
and with known sex
dsub3<-dsub2[dsub2$SEX %in% c("female", "male"),]
nrow(dsub3[is.na(dsub3$ISIZE)==F,]) # 55 individuals included in reduce datas
et

## [1] 55

# GLMM using the subset of fish with with size at birth < 8mm and with known
sex, including the same predictors as model 1, plus sex
m4<-glmmTMB(ISIZE ~ LOCATION + FOOD + SEX + MOMSZ + LOCATION:MOMSZ + (1|MOM),
         family=gaussian, data=dsub3)
summary(m4)

##  Family: gaussian  ( identity )
## Formula:
## ISIZE ~ LOCATION + FOOD + SEX + MOMSZ + LOCATION:MOMSZ + (1 |      MOM)
## Data: dsub3
##
##      AIC      BIC   logLik deviance df.resid
##     91.6    107.6    -37.8     75.6       47
##
## Random effects:
##
## Conditional model:
##  Groups   Name        Variance Std.Dev.
##  MOM      (Intercept) 0.0820   0.2864
##  Residual             0.1667   0.4082
## Number of obs: 55, groups:  MOM, 27
##
## Dispersion estimate for gaussian family (sigma^2): 0.167
##
## Conditional model:
##                       Estimate Std. Error z value Pr(>|z|)
## (Intercept)            3.61327    1.31608   2.745  0.00604 **
## LOCATIONWacissa        3.41619    1.61170   2.120  0.03404 *
## FOODLow               -0.21963    0.13230  -1.660  0.09690 .
## SEXmale               -0.02135    0.13808  -0.155  0.87712
## MOMSZ                  0.12160    0.05569   2.184  0.02900 *
## LOCATIONWacissa:MOMSZ -0.12134    0.06723  -1.805  0.07110 .
## ---
## Signif. codes:  0 '***' 0.001 '**' 0.01 '*' 0.05 '.' 0.1 ' ' 1

# diagnostic plot of residuals versus fitted values
res_m4<-resid(m4)
fitted_m4<-fitted(m4)
plot(res_m4~fitted_m4, las=1)
```

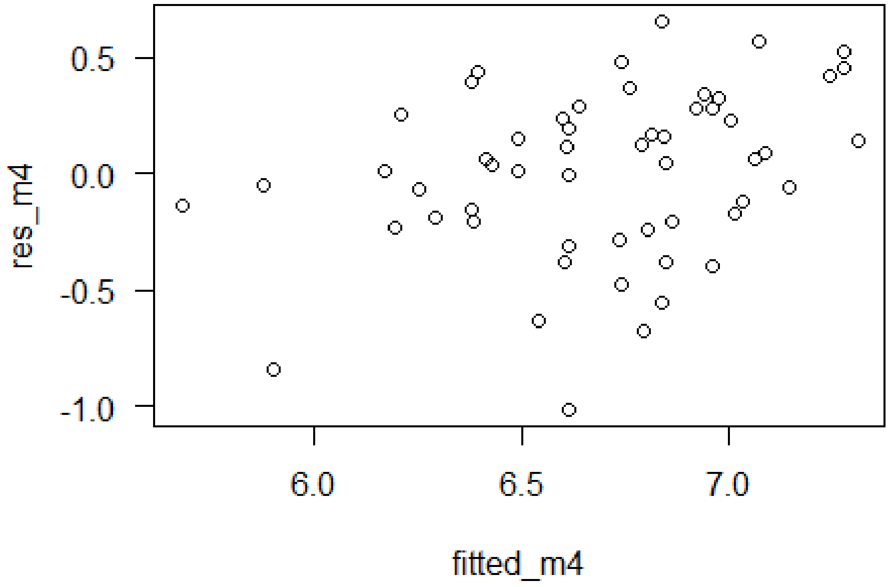


```
# obtaining a p-value for effect of maternal identity
m4a<-glmmTMB(ISIZE ~ LOCATION + FOOD + SEX + MOMSZ + LOCATION:MOMSZ,
         family=gaussian, data=dsub3)
anova(m4, m4a)

## Data: dsub3
## Models:
## m4a: ISIZE ~ LOCATION + FOOD + SEX + MOMSZ + LOCATION:MOMSZ, zi=~0, disp=~
1
## m4: ISIZE ~ LOCATION + FOOD + SEX + MOMSZ + LOCATION:MOMSZ + (1 | , zi=~0,
disp=~1
## m4:     MOM), zi=~0, disp=~1
##      Df    AIC    BIC  logLik deviance  Chisq Chi Df Pr(>Chisq)
## m4a  7 92.659 106.71 -39.329   78.659
## m4   8 91.560 107.62 -37.780   75.560 3.0985      1    0.07836 .
## ---
## Signif. codes:  0 '***' 0.001 '**' 0.01 '*' 0.05 '.' 0.1 ' ' 1
```

3.6 Figure 3

```
par(mfrow=c(1,2))
par(mar=c(4.5,4.5,1,1), xpd=F, cex=0.66)

# Figure 3a
plot(d$ISIZE~d$LOCATION, las=1, xlab="Population", ylab="", outlier.color=NA,
cex.lab=1.6, cex.axis=1.6, xaxt="n")
axis(1, at=c(1,2), labels=c("Trout P.", "Wacissa R."), mgp=c(2.5,1,0), cex.ax
```

```
is=1.6)
title(ylab="Size at birth [mm]", mgp=c(2.5,2.5,0), cex.lab=1.6)
beeswarm(d$ISIZE~d$LOCATION, pch=21, bg="lavenderblush4", cex=1.6, add=T)
text(0.6,9.6, "a", font=2, cex=2)
points(tapply(d$ISIZE, d$LOCATION, mean, na.rm=TRUE), pch=24, bg="white", cex
=2.5)

# Figure 3b
dsub1$SEX<-factor(dsub1$SEX) # remove empty level "unknown" from column "SEX"
plot(dsub1$ISIZE~dsub1$SEX, las=1, xlab="Sex", ylab="", outlier.color=NA, cex
.lab=1.6, cex.axis=1.6, xaxt="n")
axis(1, at=c(1,2), labels=c("Female", "Male"), mgp=c(2.5,1,0), cex.axis=1.6)
title(ylab="Size at birth [mm]", mgp=c(2.5,2.5,0), cex.lab=1.6)
beeswarm(dsub1$ISIZE~dsub1$SEX, bg="lavenderblush4", cex=1.6, pch=21, add=T)
text(0.6,9.6, "b", font=2, cex=2)
points(tapply(dsub1$ISIZE, dsub1$SEX, mean, na.rm=TRUE), pch=24, bg="white",
cex=2.5)
```

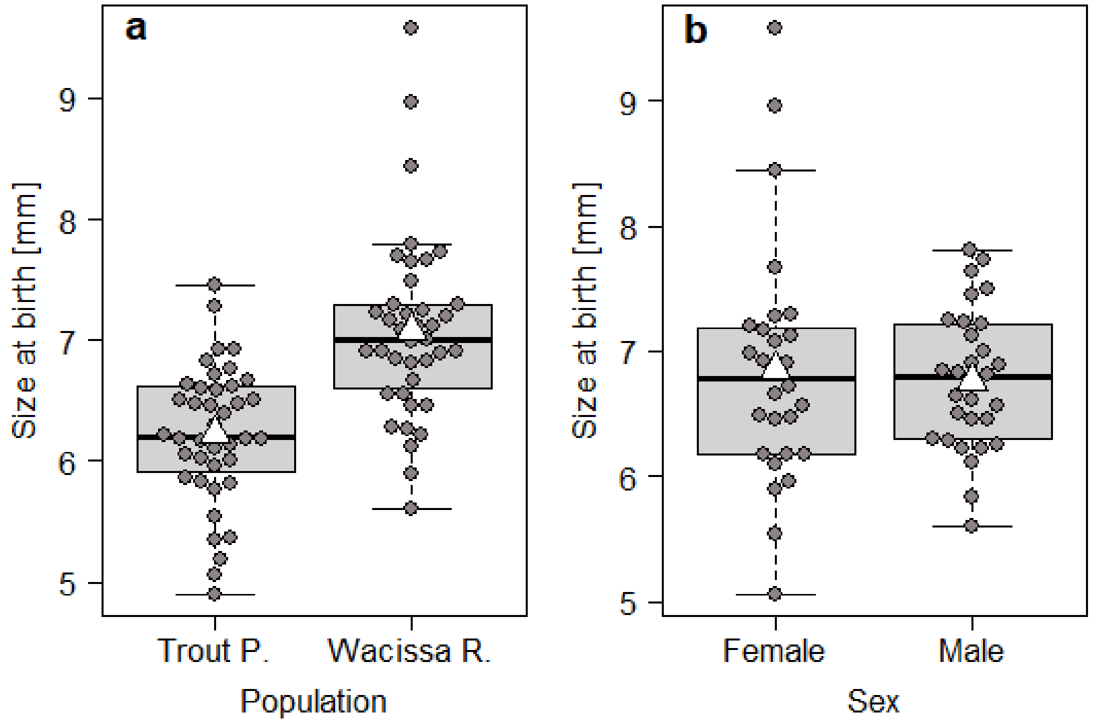

4. Analysis of survival to sexual maturity

4.1 At what age did fish die?

```
# making new columns for survival at age 14 days, 28 days, and 42 days
# use full dataset (n = 80)
d$SURV14<-ifelse(d$SURV %in% 1, "yes", "no")
d$SURV14<-ifelse(is.na(d$SZ14)==F, "yes", d$SURV14)
d$SURV14<-ifelse(is.na(d$SZ28)==F, "yes", d$SURV14)
```

```
d$SURV14<-ifelse(is.na(d$SZ42)==F, "yes", d$SURV14)
d$SURV14<-ifelse(is.na(d$SMAT)==F, "yes", d$SURV14)
d$SURV14<-ifelse(is.na(d$AGEMAT)==F, "yes", d$SURV14)

d$SURV28<-ifelse(d$SURV %in% 1, "yes", "no")
d$SURV28<-ifelse(is.na(d$SZ28)==F, "yes", d$SURV28)
d$SURV28<-ifelse(is.na(d$SZ42)==F, "yes", d$SURV28)
d$SURV28<-ifelse(is.na(d$SMAT)==F, "yes", d$SURV28)
d$SURV28<-ifelse(is.na(d$AGEMAT)==F, "yes", d$SURV28)

d$SURV42<-ifelse(d$SURV %in% 1, "yes", "no")
d$SURV42<-ifelse(is.na(d$SZ42)==F, "yes", d$SURV42)
d$SURV42<-ifelse(is.na(d$SMAT)==F, "yes", d$SURV42)
d$SURV42<-ifelse(is.na(d$AGEMAT)==F, "yes", d$SURV42)

nrow(d[d$SURV %in% 1,]) # 59 fish survived to maturity

## [1] 59

nrow(d[d$SURV42 %in% "yes",]) # 59 fish survived to age 42 days

## [1] 59

nrow(d[d$SURV28 %in% "yes",]) # 61 fish survived to age 28 days

## [1] 61

nrow(d[d$SURV14 %in% "yes",]) # 67 fish survived to age 14 days

## [1] 67

mort.rate<-c(1-(80/80), 1-(67/80), 1-(61/80), 1-(59/80))
mort.rate

## [1] 0.0000 0.1625 0.2375 0.2625
```

4.2 Identical effects of population of origin and food levels

```
table(d$SURV, d$locfood)

##
##     Trout-H Trout-L Wacissa-H Wacissa-L
##   0       3      15         0         3
##   1      17       5        20        17

# survival rate of WR fish (37/40, i.e. 92.5%) vs. TP fish (22/40, i.e. 55.0%
)
# survival rate of high-food fish (37/40, i.e. 92.5%) vs. low-food fish (22/4
0, i.e. 55.0%)
```

4.3 Model 5: survival to sexual maturity

```
nrow(d[is.na(d$SURV)==F,]) # sample size is 80 individuals

## [1] 80

# GLMM with binomial errors including the population of origin, experimental
food level, size at birth and maternal size as fixed effects, and maternal id
entity as a random effect. Note that the population-food level interaction co
uld not be included because of the mirror-image effects of population and foo
d level (see above).
m5<-glmmTMB(SURV ~ LOCATION + FOOD + ISIZE + MOMSZ + (1|MOM), family=binomial
, data=d)
summary(m5)

##  Family: binomial  ( logit )
## Formula:          SURV ~ LOCATION + FOOD + ISIZE + MOMSZ + (1 | MOM)
## Data: d
## 
##      AIC      BIC   logLik deviance df.resid
##     65.4     79.7    -26.7     53.4       73
## 
## Random effects:
## 
## Conditional model:
##  Groups Name        Variance  Std.Dev.
##  MOM    (Intercept) 1.108e-08 0.0001053
## Number of obs: 79, groups:  MOM, 31
## 
## Conditional model:
##                   Estimate Std. Error z value Pr(>|z|)
## (Intercept)        -5.6215     5.6517  -0.995 0.319902
## LOCATIONWacissa     2.9516     1.0157   2.906 0.003660 **
## FOODLow            -2.9369     0.8231  -3.568 0.000359 ***
## ISIZE               0.1596     0.6128   0.260 0.794532
## MOMSZ               0.2798     0.1678   1.667 0.095451 .
## ---
## Signif. codes:  0 '***' 0.001 '**' 0.01 '*' 0.05 '.' 0.1 ' ' 1

# diagnostic plot of residuals versus fitted values
res_m5<-resid(m5)
fitted_m5<-fitted(m5)
plot(res_m5~fitted_m5, las=1)
```

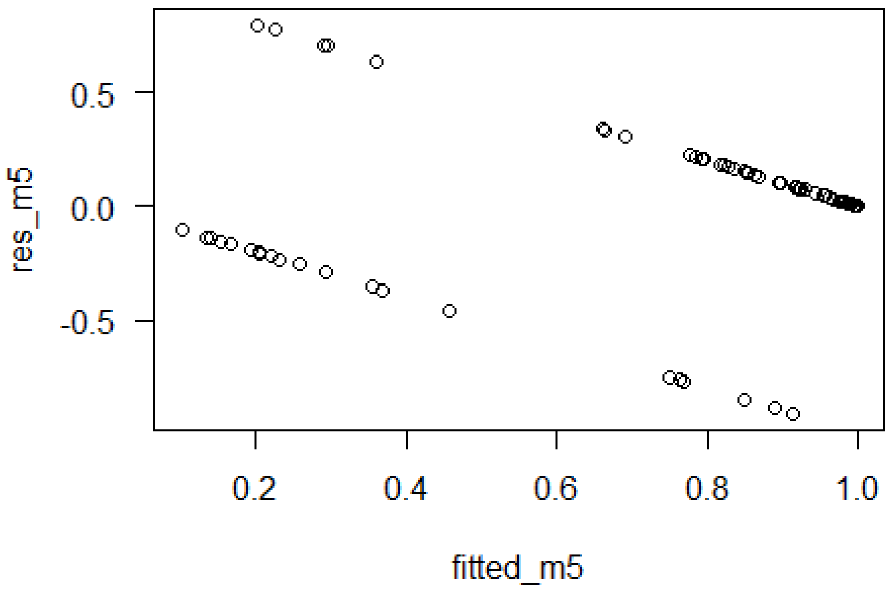


```
# obtaining a p-value for effect of maternal identity
m5a<-glmmTMB(SURV ~ LOCATION + FOOD + ISIZE + MOMSZ, family=binomial, data=d)
anova(m5, m5a)

## Data: d
## Models:
## m5a: SURV ~ LOCATION + FOOD + ISIZE + MOMSZ, zi=~0, disp=~1
## m5: SURV ~ LOCATION + FOOD + ISIZE + MOMSZ + (1 | MOM), zi=~0, disp=~1
##     Df    AIC    BIC logLik deviance Chisq Chi Df Pr(>Chisq)
## m5a  5 63.439 75.286 -26.72   53.439
## m5   6 65.439 79.656 -26.72   53.439     0      1          1
```

4.4 Model 6: survival to sexual maturity (test effect of size at birth once population is removed as a predictor)

```
# Same GLMM as in model 5, except that population of origin is removed from t
he list of predictors.
m6<-glmmTMB(SURV ~ FOOD + ISIZE + MOMSZ + (1|MOM), data=d, family=binomial)
summary(m6)

##  Family: binomial  ( logit )
## Formula:          SURV ~ FOOD + ISIZE + MOMSZ + (1 | MOM)
## Data: d
##
##      AIC      BIC   logLik deviance df.resid
##     75.0     86.9    -32.5     65.0       74
##
```

```
## Random effects:
##
## Conditional model:
##  Groups Name        Variance  Std.Dev.
##  MOM    (Intercept) 1.627e-07 0.0004034
## Number of obs: 79, groups:  MOM, 31
##
## Conditional model:
##             Estimate Std. Error z value Pr(>|z|)
## (Intercept) -10.9480     4.5643  -2.399  0.01646 *
## FOODLow      -2.2509     0.7308  -3.080  0.00207 **
## ISIZE         1.2156     0.4785   2.541  0.01106 *
## MOMSZ         0.2412     0.1346   1.792  0.07317 .
## ---
## Signif. codes:  0 '***' 0.001 '**' 0.01 '*' 0.05 '.' 0.1 ' ' 1
```

4.5 Chi-square test for survival to sexual maturity

```
tbl<-table(d$SURV, d$locfood)
tbl

##
##     Trout-H Trout-L Wacissa-H Wacissa-L
##   0       3      15         0         3
##   1      17       5        20        17

# performing a Pearson's Chi-squared test
chisq<-chisq.test(tbl)
chisq

##
##  Pearson's Chi-squared test
##
## data:  tbl
## X-squared = 34.286, df = 3, p-value = 1.724e-07

# computing the relative contribution of each cell in the contingency table t
o the total Chi-square score
round(chisq$expected,2)

##
##     Trout-H Trout-L Wacissa-H Wacissa-L
##   0    5.25    5.25      5.25      5.25
##   1   14.75   14.75     14.75     14.75

round(chisq$residuals, 3)

##
##     Trout-H Trout-L Wacissa-H Wacissa-L
##   0  -0.982   4.255    -2.291    -0.982
##   1   0.586  -2.539     1.367     0.586
```

```
contrib<-100*chisq$residuals^2/chisq$statistic
round(contrib, 3)

##
##     Trout-H Trout-L Wacissa-H Wacissa-L
##   0   2.812  52.813    15.312     2.812
##   1   1.001  18.798     5.450     1.001
```

4.6 Computing binomial standard errors for survival rates

Shown in Figure 4a.

```
survivors<-c(17, 20, 5, 17)
n<-20

p<-survivors/n
se<-sqrt((p*(1-p))/(n-1))

prop<-survivors/n
```

4.7 Figure 4

```
# adjust levels of column "survloc" for Figure 4b
levels(d$survloc)

## [1] "Tr-alive" "Tr-dead"  "Wa-alive" "Wa-dead"

d$survloc<-factor(d$survloc, levels=c("Tr-dead", "Wa-dead", "Tr-alive", "Wa-a
live"))

par(mfrow=c(1,3))
par(mar=c(4.5,5,1,1), xpd=T)

# Figure 4a
a<-barplot(height=cbind(a = c(0.85, 1.00),
                        b = c(0.25, 0.85)),
           beside=TRUE,
             col=c("green1","lightskyblue"),
             xlab="", yaxt="n", cex.axis=1.6, cex.lab=1.6,cex.names=1.6, las=
1,
             names.arg=c("High", "Low"),
               space=c(0, 0.5), ylim=c(0, 1.2))

title(ylab="Survival to maturity", mgp=c(3, 1.5, 0), cex.lab=1.6)
axis(2, at=c(0, 0.2, 0.4, 0.6, 0.8, 1), cex.axis=1.6, las=1)
title(xlab="Food level", mgp=c(3, 1.5, 0), cex.lab=1.6)

legend("topright", legend=c("Trout P.", "Wacissa R."), fill=c("green1", "ligh
tskyblue"),
```

```
col=c("green1", "lightskyblue"), bty="n", cex=1.6, inset=c(-0.045, 0.0))
text(-0.8, 1.154, "a", font=2, cex=2)

segments(a, prop - se, a, prop + se, lwd = 1.5)

arrows(a, prop - se, a, prop + se, lwd = 1.5, angle = 90,
       code = 3, length = 0.05)

# Figure 4b
plot(d$ISIZE~d$survloc, las=1, xlab="Survival to maturity", ylab="", col=c("g
reen1", "lightskyblue",
"green1", "lightskyblue"), outlier.color=NA, cex.lab=1.6, cex.axis=1.6, xaxt=
"n")
axis(1, at=c(1.5,3.5), labels=c("No", "Yes"), mgp=c(2.5,1,0), cex.axis=1.6)
title(ylab="Size at birth [mm]", mgp=c(2.5,2.5,0), cex.lab=1.6)
beeswarm(d$ISIZE~d$survloc, pch=21, bg=c("green1", "lightskyblue",
"green1", "lightskyblue"), cex=1.6, add=T)
text(0.6,9.6, "b", font=2, cex=2)
legend(0.7, 9.9, legend=c("Trout P.", "Wacissa R."), pt.bg=c("green1", "light
skyblue"),
col=c("black", "black"), bty="n", pch=21, cex=1.6, pt.cex=1.6)
rect(0.82, 9.05, 3.1, 9.77)
points(tapply(d$ISIZE, d$survloc, mean, na.rm=TRUE), pch=24, bg="white", cex=
2.5)

# Figure 4c
plot(d$MOMSZ~d$SURV, las=1, xlab="Survival to maturity", ylab="", outlier.col
or=NA, cex.lab=1.6, cex.axis=1.6, xaxt="n")
axis(1, at=c(1,2), labels=c("No", "Yes"), mgp=c(2.5,1,0), cex.axis=1.6)
title(ylab="Mother's size [mm]", mgp=c(2.7,2.5,0), cex.lab=1.6)
beeswarm(d$MOMSZ~d$SURV, pch=21, bg="lavenderblush4", cex=1.6, add=T)
text(0.6,29.8, "c", font=2, cex=2)
points(tapply(d$MOMSZ, d$SURV, mean, na.rm=TRUE), pch=24, bg="white", cex=2.5
)
```

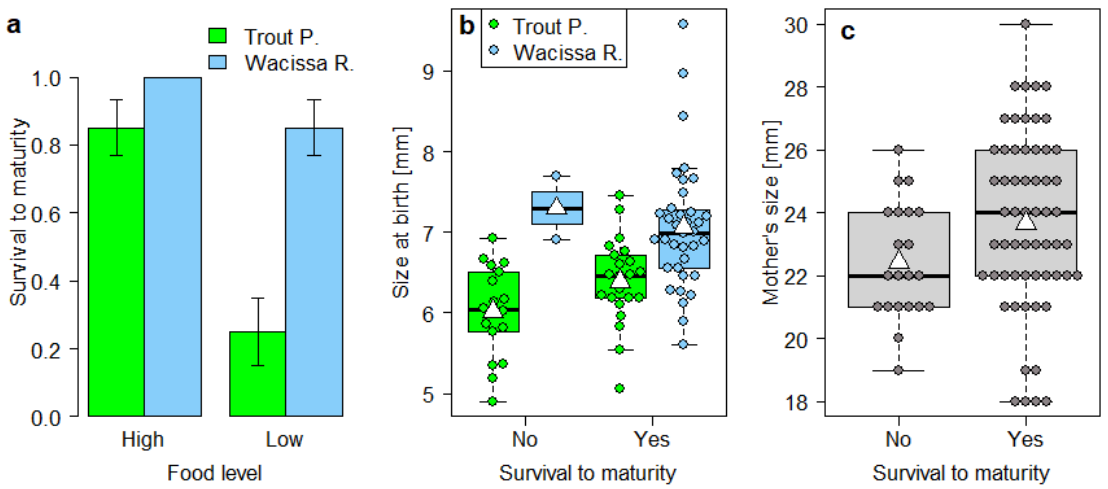

5. Analysis of size at 14 days

5.1 Distribution of size at 14 days

```
# exclude fish without data on their size at 14 days
dsub4<-d[is.na(d$SZ14)==F,]
nrow(dsub4) # 65 individuals

## [1] 65

# two-sided Kolmogorov-Smirnov test, showing no significant deviation from no
rmality
ks.test(dsub4$SZ14, pnorm, mean(dsub4$SZ14), sd(dsub4$SZ14))
##  One-sample Kolmogorov-Smirnov test
##
## data:  dsub4$SZ14
## D = 0.11701, p-value = 0.3357
## alternative hypothesis: two-sided

# histogram and quantile-quantile plot, also indicating that data are approxi
mately normally distributed
par(mfrow=c(1,2))
hist(dsub4$SZ14, breaks=50, main="", xlab="Size at 14 days[mm]", las=1)
qqnorm(dsub4$SZ14, pch = 1, frame = FALSE, main="", las=1)
qqline(dsub4$SZ14, col = "steelblue", lwd = 2)
```

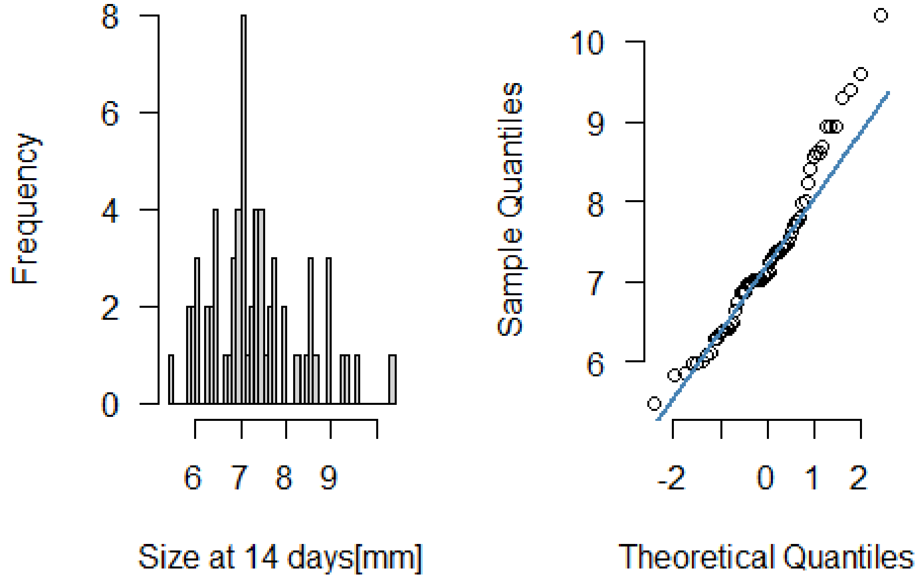

5.2 Computing proportional differences between populations and food levels

```
tapply(dsub4$SZ14, dsub4$LOCATION, mean)

##    Trout  Wacissa
## 6.784444 7.711316

7.711316/6.784444

## [1] 1.136617

tapply(dsub4$SZ14, dsub4$FOOD, mean)

##     High      Low
## 7.713056 6.846207

7.713056/6.846207

## [1] 1.126617
```

5.3 Model 7: size at 14 days

```
# GLMM with Gaussian errors including the population of origin, experimental
food level, their interaction, maternal size, and the size at birth as fixed
effects, and maternal identity as a random effect
m7<-glmmTMB(SZ14 ~ LOCATION + FOOD + LOCATION:FOOD + MOMSZ + ISIZE + (1|MOM),
data=dsub4)
summary(m7)

##  Family: gaussian  ( identity )
## Formula:
## SZ14 ~ LOCATION + FOOD + LOCATION:FOOD + MOMSZ + ISIZE + (1 |      MOM)
## Data: dsub4
##
##      AIC      BIC   logLik deviance df.resid
##    100.1    117.5    -42.0     84.1       57
##
## Random effects:
##
## Conditional model:
##  Groups   Name        Variance Std.Dev.
##  MOM      (Intercept) 0.06151  0.248
##  Residual             0.16480  0.406
## Number of obs: 65, groups:  MOM, 28
##
## Dispersion estimate for gaussian family (sigma^2): 0.165
##
## Conditional model:
##                            Estimate Std. Error z value Pr(>|z|)
## (Intercept)                 0.19113    0.88518   0.216  0.82905
## LOCATIONWacissa             0.41512    0.18497   2.244  0.02482 *
```

```
## FOODLow                    -0.48791    0.17681  -2.759  0.00579 **
## MOMSZ                       0.05443    0.02649   2.054  0.03996 *
## ISIZE                       0.87513    0.09697   9.025  < 2e-16 ***
## LOCATIONWacissa:FOODLow -0.20286    0.22029  -0.921  0.35712
## ---
## Signif. codes:  0 '***' 0.001 '**' 0.01 '*' 0.05 '.' 0.1 ' ' 1
```

```
# diagnostic plot of residuals versus fitted values
res_m7<-resid(m7)
fitted_m7<-fitted(m7)
plot(res_m7~fitted_m7, las=1)
```

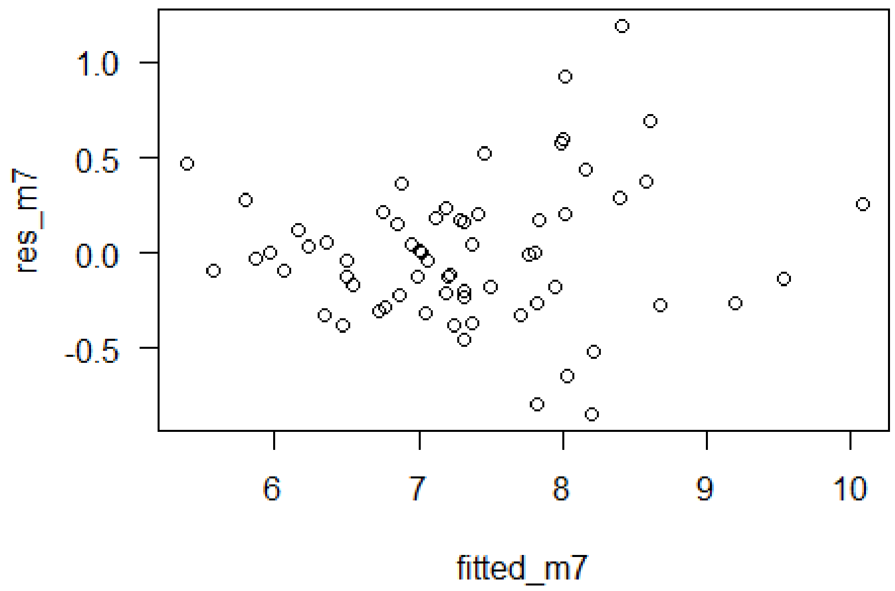


```
# obtaining a p-value for the effect of maternal identity
m7a<-glmmTMB(SZ14 ~ LOCATION + FOOD + LOCATION:FOOD + MOMSZ + ISIZE, data=dsu
b4)
anova(m7, m7a)
```

```
## Data: dsub4
## Models:
## m7a: SZ14 ~ LOCATION + FOOD + LOCATION:FOOD + MOMSZ + ISIZE, zi=~0, disp=~
1
## m7: SZ14 ~ LOCATION + FOOD + LOCATION:FOOD + MOMSZ + ISIZE + (1 | , zi=~0,
disp=~1
## m7:     MOM), zi=~0, disp=~1
##      Df    AIC    BIC  logLik deviance  Chisq Chi Df Pr(>Chisq)
## m7a   7 100.06 115.28 -43.032   86.064
## m7    8 100.06 117.45 -42.030   84.060 2.0043      1     0.1569
```

5.4 Model 8: size at 14 days (test of influence of outliers)

This model excludes the four fish with very large sizes at 14 days, identified as potential outliers in the QQ-plot.

```
# making a new dataset that only contains fish with a size at 14 days < 9mm
dsub5<-dsub4[dsub4$SZ14 < 9.00,]
nrow(dsub5) # 61 individuals

## [1] 61

# GLMM using the subset of fish with size at 14 days < 9mm, including the sam
e predictors as model 7
m8<-glmmTMB(SZ14 ~ LOCATION + FOOD + LOCATION:FOOD + MOMSZ + ISIZE + (1|MOM),
data=dsub5)
summary(m8)

##  Family: gaussian  ( identity )
## Formula:
## SZ14 ~ LOCATION + FOOD + LOCATION:FOOD + MOMSZ + ISIZE + (1 |      MOM)
## Data: dsub5
##
##      AIC      BIC   logLik deviance df.resid
##     77.8     94.7    -30.9     61.8       53
##
## Random effects:
##
## Conditional model:
##  Groups   Name        Variance Std.Dev.
##  MOM      (Intercept) 0.09438  0.3072
##  Residual             0.09907  0.3148
## Number of obs: 61, groups:  MOM, 27
##
## Dispersion estimate for gaussian family (sigma^2): 0.0991
##
## Conditional model:
##                         Estimate Std. Error z value Pr(>|z|)
## (Intercept)              1.60870    0.91378   1.760 0.078324 .
## LOCATIONWacissa          0.45728    0.18127   2.523 0.011649 *
## FOODLow                 -0.49913    0.14437  -3.457 0.000546 ***
## MOMSZ                    0.02525    0.03006   0.840 0.400822
## ISIZE                    0.75240    0.09918   7.586 3.29e-14 ***
## LOCATIONWacissa:FOODLow -0.09369    0.18492  -0.507 0.612416
## ---
## Signif. codes:  0 '***' 0.001 '**' 0.01 '*' 0.05 '.' 0.1 ' ' 1

# diagnostic plot of residuals versus fitted values
res_m8<-resid(m8)
fitted_m8<-fitted(m8)
plot(res_m8~fitted_m8, las=1)
```

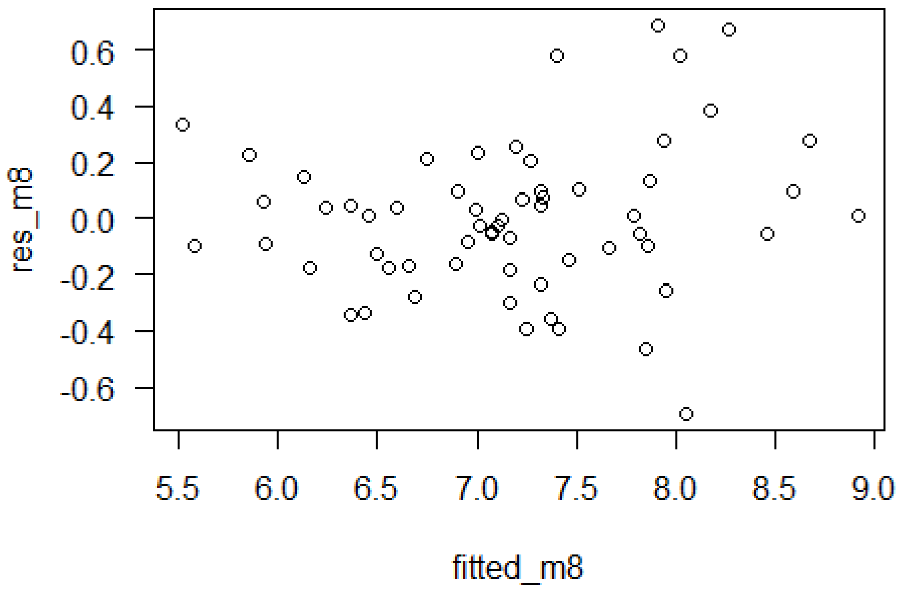


```
# obtaining a p-value for the effect of maternal identity
m8a<-glmmTMB(SZ14 ~ LOCATION + FOOD + LOCATION:FOOD + MOMSZ + ISIZE, data=dsu
b5)
anova(m8, m8a)

## Data: dsub5
## Models:
## m8a: SZ14 ~ LOCATION + FOOD + LOCATION:FOOD + MOMSZ + ISIZE, zi=~0, disp=~
1
## m8: SZ14 ~ LOCATION + FOOD + LOCATION:FOOD + MOMSZ + ISIZE + (1 | , zi=~0,
disp=~1
## m8:     MOM), zi=~0, disp=~1
##     Df    AIC    BIC  logLik deviance  Chisq Chi Df Pr(>Chisq)
## m8a  7 84.121 98.897 -35.061   70.121
## m8   8 77.819 94.706 -30.910   61.819 8.3022      1    0.00396 **
## ---
## Signif. codes:  0 '***' 0.001 '**' 0.01 '*' 0.05 '.' 0.1 ' ' 1
```

5.5 Figure 5

```
# save partial regression coefficients for Figure 5b
m7.coef<-as.data.frame(summary(m7)$coefficients[1])
m7_inter<-m7.coef[1,1]
m7_locWR<-m7.coef[2,1]
m7_foodL<-m7.coef[3,1]
m7_momsz<-m7.coef[4,1]
m7_isize<-m7.coef[5,1]
```

```
m7_WRL<-m7.coef[6,1]

# save ranges of size at birth for all experimental groups of fish, for Figur
e 5b
isizes_WRhigh<-seq(min(dsub4$ISIZE[dsub4$locfood %in% "Wacissa-H"]), max(dsub
4$ISIZE[dsub4$locfood %in% "Wacissa-H"]), 0.1)
isizes_WRlow<-seq(min(dsub4$ISIZE[dsub4$locfood %in% "Wacissa-L"]), max(dsub4
$ISIZE[dsub4$locfood %in% "Wacissa-L"]), 0.1)
isizes_TPhigh<-seq(min(dsub4$ISIZE[dsub4$locfood %in% "Trout-H"]), max(dsub4$
ISIZE[dsub4$locfood %in% "Trout-H"]), 0.1)
isizes_TPlow<-seq(min(dsub4$ISIZE[dsub4$locfood %in% "Trout-L"]), max(dsub4$I
SIZE[dsub4$locfood %in% "Trout-L"]), 0.1)

par(mfrow=c(1,3))
par(mar=c(4.5,4.5,1,1), xpd=F)

# Figure 5a
plot(dsub4$SZ14~dsub4$locfood, las=1, xlab="Population", ylab="", col=c("yell
ow", "indianred1", "yellow1","indianred1"), outlier.color=NA, cex.lab=1.6, ce
x.axis=1.6, xaxt="n")
axis(1, at=c(1.5,3.5), labels=c("Trout P.", "Wacissa R."), mgp=c(2.5,1,0), ce
x.axis=1.6)
title(ylab="Size at 14 days [mm]", mgp=c(2.5,2.5,0), cex.lab=1.6)
beeswarm(dsub4$SZ14~dsub4$locfood, pch=21, bg=c("yellow1", "indianred1", "yel
low1", "indianred1"), cex=1.6, add=T)
text(0.6, 10.3, "a", font=2, cex=2)
legend(0.7, 10.65, legend=c("High food", "Low food"), pt.bg=c("yellow1", "ind
ianred1"),
col=c("black", "black"), bty="n", pch=21, cex=1.6, pt.cex=1.6, inset=c(0, -0.
02))
rect(0.8, 9.8, 2.7, 13)
points(tapply(dsub4$SZ14, dsub4$locfood, mean), pch=24, bg="white", cex=2.5)

# Figure 5b
plot(dsub4$SZ14~dsub4$ISIZE, las=1, xlab="Size at birth [mm]", ylab="", cex.l
ab=1.6, cex.axis=1.6, cex=1.6, col="white")
title(ylab="Size at 14 days [mm]", mgp=c(2.5,2.5,0), cex.lab=1.6)
text(5.2, 10.3, "b", font=2, cex=2)

points(dsub4$SZ14[dsub4$locfood %in% "Wacissa-H"]~dsub4$ISIZE[dsub4$locfood %
in% "Wacissa-H"], pch=21, bg="green1", cex=1.6)
points(dsub4$SZ14[dsub4$locfood %in% "Wacissa-L"]~dsub4$ISIZE[dsub4$locfood %
in% "Wacissa-L"], pch=21, bg="lightskyblue1", cex=1.6)
points(dsub4$SZ14[dsub4$locfood %in% "Trout-H"]~dsub4$ISIZE[dsub4$locfood %in
% "Trout-H"], pch=21, bg="yellow1", cex=1.6)
points(dsub4$SZ14[dsub4$locfood %in% "Trout-L"]~dsub4$ISIZE[dsub4$locfood %in
% "Trout-L"], pch=21, bg="indianred1", cex=1.6)

lines(isizes_WRhigh, m7_inter + m7_locWR*1 + m7_foodL*0 + m7_momsz*(mean(dsub
4$MOMSZ[dsub4$locfood %in% "Wacissa-H"])) + m7_isize*isizes_WRhigh + m7_WRL*1
```

```
*0, lwd=1.8, col="green1")
lines(isizes_WRlow, m7_inter + m7_locWR*1 + m7_foodL*1 + m7_momsz*(mean(dsub4
$MOMSZ[dsub4$locfood %in% "Wacissa-L"])) + m7_isize*isizes_WRlow + m7_WRL*1*1
, lwd=1.8, col="lightskyblue4")
lines(isizes_TPhigh, m7_inter + m7_locWR*0 + m7_foodL*0 + m7_momsz*(mean(dsub
4$MOMSZ[dsub4$locfood %in% "Trout-H"])) + m7_isize*isizes_TPhigh + m7_WRL*0*0
, lwd=1.8, col="yellow4")
lines(isizes_TPlow, m7_inter + m7_locWR*0 + m7_foodL*1 + m7_momsz*(mean(dsub4
$MOMSZ[dsub4$locfood %in% "Trout-L"])) + m7_isize*isizes_TPlow + m7_WRL*0*1,
lwd=1.8, col="indianred3")

legend("bottomright", legend=c("TP-High food", "TP-Low food", "WR-High food",
"WR-Low food"), pt.bg=c("yellow1", "indianred1", "green1", "lightskyblue"),
col=c("black", "black"), bty="n", pch=21, cex=1.6, pt.cex=1.6, inset=c(0, -0.
02))
rect(6.77, 5, 10, 6.66)

# Figure 5c
plot(dsub4$SZ14~dsub4$SEX, las=1, xlab="Sex", ylab="", pch=21, outlier.color=
NA, cex.lab=1.6, cex.axis=1.6, xaxt="n")
axis(1, at=c(1,2,3), labels=c("Female", "Male", "Unknown"), mgp=c(2.5,1,0), c
ex.axis=1.6)
title(ylab="Size at 14 days [mm]", mgp=c(2.5,2.5,0), cex.lab=1.6)
beeswarm(dsub4$SZ14~dsub4$SEX, bg="lavenderblush4", cex=1.6, pch=21, add=T)
text(0.6, 10.3, "c", font=2, cex=2)
points(tapply(dsub4$SZ14, dsub4$SEX, mean), pch=24, bg="white", cex=2.5)
```

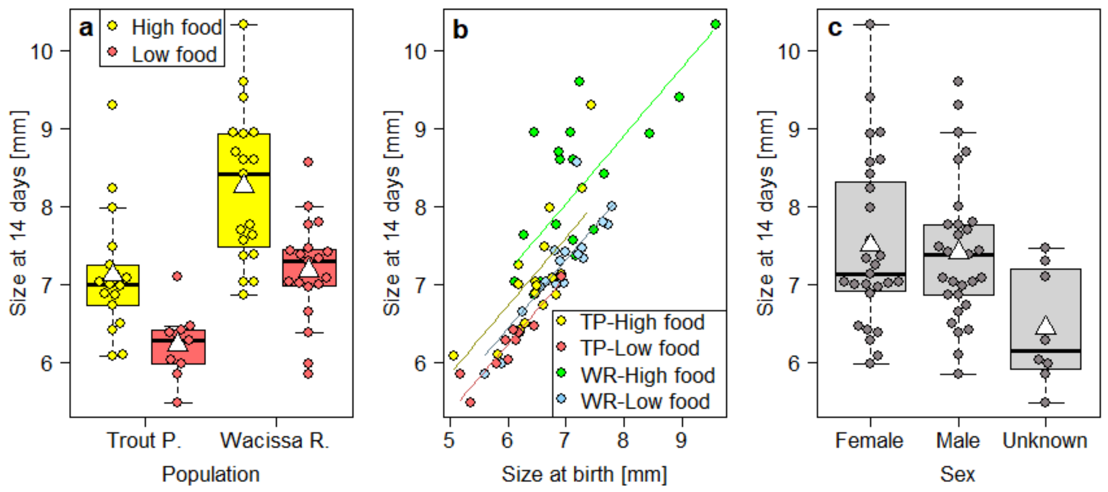

6. Analysis of size as a juvenile and at maturity

6.1 Data preparation

```
# exclude fish without data on their size at 14 days, 28 days, 42 days, and m
aturity
dsub6<-d[is.na(d$SZ14)==F,]
```

```
dsub6<-dsub6[is.na(dsub6$SZ28)==F,]
dsub6<-dsub6[is.na(dsub6$SZ42)==F,]
dsub6<-dsub6[is.na(dsub6$SMAT)==F,]
nrow(dsub6) # 54 individuals

## [1] 54

# re-organise data to be in the long format (i.e., one measurement per row)
sz14<-dsub6[,c(1:4, 6, 10:21, 7)]
sz28<-dsub6[,c(1:4, 6, 10:21, 8)]
sz42<-dsub6[,c(1:4, 6, 10:21, 9)]
smat<-dsub6[,c(1:4, 6, 10:21, 5)]
colnames(sz14)[18]<-"SIZE"
colnames(sz28)[18]<-"SIZE"
colnames(sz42)[18]<-"SIZE"
colnames(smat)[18]<-"SIZE"
sz14$AGE<-1
sz28$AGE<-2
sz42$AGE<-3
smat$AGE<-4
dlong<-rbind(sz14, sz28, sz42, smat)
nrow(dlong) # 216 rows (i.e., 4 measurements x 54 individuals)

## [1] 216

dlong$AGE<-as.factor(dlong$AGE)
```

6.2 Distribution of size as a juvenile and at maturity

```
# two-sided Kolmogorov-Smirnov test, showing no significant deviation from no
rmality
ks.test(dlong$SIZE, pnorm, mean(dlong$SIZE), sd(dlong$SIZE))
##  One-sample Kolmogorov-Smirnov test
##
## data:  dlong$SIZE
## D = 0.068381, p-value = 0.2647
## alternative hypothesis: two-sided

# histogram and quantile-quantile plot, also indicating that data are approxi
mately normally distributed
par(mfrow=c(1,2))
hist(dlong$SIZE, breaks=50, main="", xlab="Size [mm]", las=1)
qqnorm(dlong$SIZE, pch = 1, frame = FALSE, main="", las=1)
qqline(dlong$SIZE, col = "steelblue", lwd = 2)
```

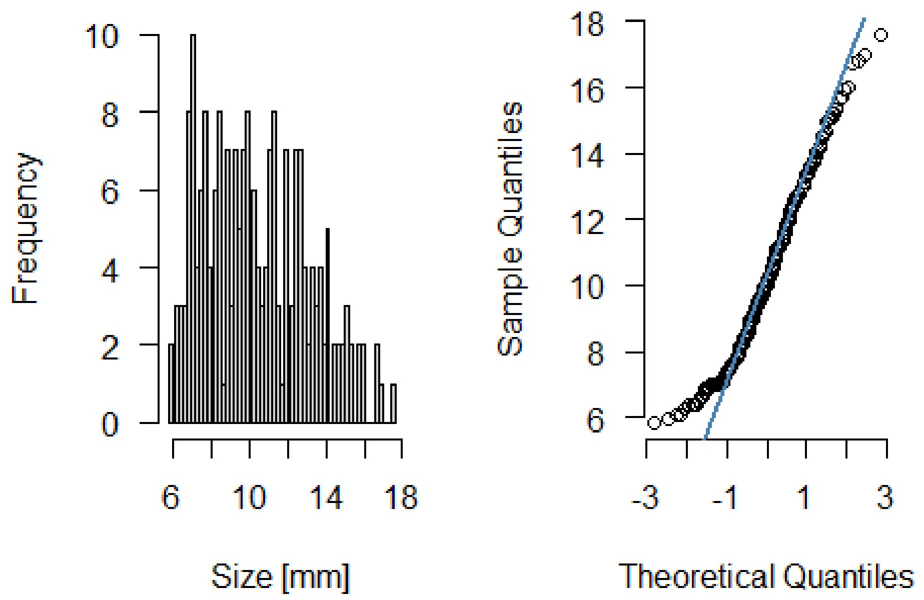

6.3 Model 9: size as a juvenile and at maturity

```
# analysis of variance with repeated measures of juvenile sizes and size at s
exual maturity
m9<-aov(SIZE ~ LOCATION + FOOD + AGE + AGE:LOCATION + AGE:FOOD + SEX + SEX:AG
E + ISIZE + ISIZE:AGE + MOMSZ + MOM + Error(FISHID), data=dlong)
summary(m9)

##
## Error: FISHID
##           Df Sum Sq Mean Sq F value   Pr(>F)    
## LOCATION   1  31.75   31.75   9.840  0.00447 ** 
## FOOD       1 130.84  130.84  40.547 1.39e-06 ***
## SEX        1   1.63    1.63   0.506  0.48354    
## ISIZE      1  31.60   31.60   9.793  0.00455 ** 
## MOMSZ      1  10.11   10.11   3.132  0.08949 .  
## MOM       24  89.34    3.72   1.154  0.36458    
## Residuals 24  77.45    3.23                     
## ---
## Signif. codes:  0 '***' 0.001 '**' 0.01 '*' 0.05 '.' 0.1 ' ' 1
##
## Error: Within
##               Df Sum Sq Mean Sq F value   Pr(>F)    
## AGE            3  974.3   324.8 325.052  < 2e-16 ***
## LOCATION:AGE   3    0.6     0.2   0.192  0.90151    
## FOOD:AGE       3   16.2     5.4   5.402  0.00149 ** 
## AGE:SEX        3   66.6    22.2  22.217 6.39e-12 ***
```

```
## AGE:ISIZE      3   10.5      3.5   3.489  0.01738 *
## Residuals    147  146.9      1.0
## ---
## Signif. codes:  0 '***' 0.001 '**' 0.01 '*' 0.05 '.' 0.1 ' ' 1

summary(m9)[[2]][[1]][[2]] # sum of squares for within-fish effects with more
decimal places

## [1] 974.3211204   0.5764185  16.1927509  66.5934335  10.4569582 146.874068
5

summary(m9)[[2]][[1]][[3]] # mean squares for within-fish effects with more d
ecimal places

## [1] 324.7737068   0.1921395   5.3975836  22.1978112   3.4856527   0.999143
3

# diagnostic plot of residuals versus fitted values
res_m9<-residuals.aovlist(m9)
fit_m9<-fitted.aovlist(m9)
plot(fit_m9, res_m9, las=1)
```

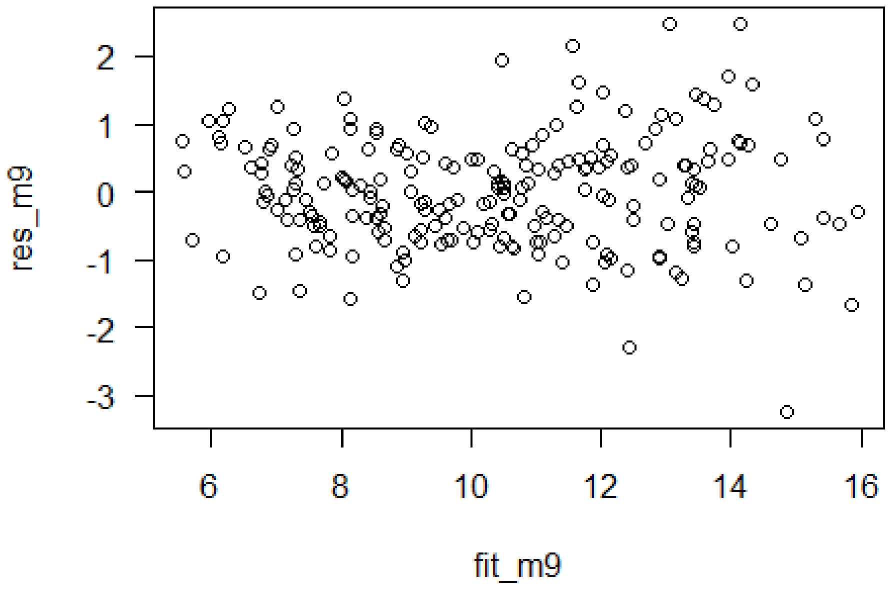

6.4 Preparations for figure 6

```
# minimum and maximum size of fish
min<-min(dlong$SIZE)
max<-max(dlong$SIZE)+1
# new column APF (age-pop-food) to sort data according to the age when fish w
```

```
ere measured, their # population and the experimental food level
dlong$APF<-ifelse(dlong$AGE %in% 1 & dlong$LOCATION %in% "Trout" & dlong$FOOD
%in% "High", 1, NA)
dlong$APF<-ifelse(dlong$AGE %in% 1 & dlong$LOCATION %in% "Trout" & dlong$FOOD
%in% "Low", 2, dlong$APF)
dlong$APF<-ifelse(dlong$AGE %in% 1 & dlong$LOCATION %in% "Wacissa" & dlong$FO
OD %in% "High", 3, dlong$APF)
dlong$APF<-ifelse(dlong$AGE %in% 1 & dlong$LOCATION %in% "Wacissa" & dlong$FO
OD %in% "Low", 4, dlong$APF)

dlong$APF<-ifelse(dlong$AGE %in% 2 & dlong$LOCATION %in% "Trout" & dlong$FOOD
%in% "High", 5, dlong$APF)
dlong$APF<-ifelse(dlong$AGE %in% 2 & dlong$LOCATION %in% "Trout" & dlong$FOOD
%in% "Low", 6, dlong$APF)
dlong$APF<-ifelse(dlong$AGE %in% 2 & dlong$LOCATION %in% "Wacissa" & dlong$FO
OD %in% "High", 7, dlong$APF)
dlong$APF<-ifelse(dlong$AGE %in% 2 & dlong$LOCATION %in% "Wacissa" & dlong$FO
OD %in% "Low", 8, dlong$APF)

dlong$APF<-ifelse(dlong$AGE %in% 3 & dlong$LOCATION %in% "Trout" & dlong$FOOD
%in% "High", 9, dlong$APF)
dlong$APF<-ifelse(dlong$AGE %in% 3 & dlong$LOCATION %in% "Trout" & dlong$FOOD
%in% "Low", 10, dlong$APF)
dlong$APF<-ifelse(dlong$AGE %in% 3 & dlong$LOCATION %in% "Wacissa" & dlong$FO
OD %in% "High", 11, dlong$APF)
dlong$APF<-ifelse(dlong$AGE %in% 3 & dlong$LOCATION %in% "Wacissa" & dlong$FO
OD %in% "Low", 12, dlong$APF)

dlong$APF<-ifelse(dlong$AGE %in% 4 & dlong$LOCATION %in% "Trout" & dlong$FOOD
%in% "High", 13, dlong$APF)
dlong$APF<-ifelse(dlong$AGE %in% 4 & dlong$LOCATION %in% "Trout" & dlong$FOOD
%in% "Low", 14, dlong$APF)
dlong$APF<-ifelse(dlong$AGE %in% 4 & dlong$LOCATION %in% "Wacissa" & dlong$FO
OD %in% "High", 15, dlong$APF)
dlong$APF<-ifelse(dlong$AGE %in% 4 & dlong$LOCATION %in% "Wacissa" & dlong$FO
OD %in% "Low", 16, dlong$APF)

# new column AGESEX to sort data according to the age when fish were measured
and their sex
dlong$AGESEX<-ifelse(dlong$AGE %in% 1 & dlong$SEX %in% "female", 1, NA)
dlong$AGESEX<-ifelse(dlong$AGE %in% 1 & dlong$SEX %in% "male", 2, dlong$AGESE
X)
dlong$AGESEX<-ifelse(dlong$AGE %in% 2 & dlong$SEX %in% "female", 3, dlong$AGE
SEX)
dlong$AGESEX<-ifelse(dlong$AGE %in% 2 & dlong$SEX %in% "male", 4, dlong$AGESE
X)
dlong$AGESEX<-ifelse(dlong$AGE %in% 3 & dlong$SEX %in% "female", 5, dlong$AGE
SEX)
dlong$AGESEX<-ifelse(dlong$AGE %in% 3 & dlong$SEX %in% "male", 6, dlong$AGESE
X)
```

```
dlong$AGESEX<-ifelse(dlong$AGE %in% 4 & dlong$SEX %in% "female", 7, dlong$AGE
SEX)
dlong$AGESEX<-ifelse(dlong$AGE %in% 4 & dlong$SEX %in% "male", 8, dlong$AGESE
X)
table(dlong$AGESEX, dlong$AGE)

##
##       1  2  3  4
##    1 25  0  0  0
##    2 29  0  0  0
##    3  0 25  0  0
##    4  0 29  0  0
##    5  0  0 25  0
##    6  0  0 29  0
##    7  0  0  0 25
##    8  0  0  0 29

table(dlong$AGESEX, dlong$SEX)

##
##      female male unknown
##    1     25    0       0
##    2      0   29       0
##    3     25    0       0
##    4      0   29       0
##    5     25    0       0
##    6      0   29       0
##    7     25    0       0
##    8      0   29       0
```

6.5 Figure 6

```
par(mar=c(4.5,4.5,1,1), xpd=F, cex=0.66)

# Figure 6a
boxplot(dlong$SIZE~dlong$APF, xlab="", col=rep(c("yellow1", "indianred1", "gr
een1", "lightskyblue"), 4),
ylim=c(min, max), at=c(0.5,1.5,2.5,3.5, 5.5,6.5,7.5,8.5, 10.5,11.5,12.5,13.5,
15.5,16.5,17.5,18.5), xaxt="n", ylab="Size [mm]",
las=1, cex.axis=1.6, cex.lab=1.6, xlim=c(0,19), outlier.color=NA, staplelwd=2
)
beeswarm(dlong$SIZE~dlong$APF, pch=21, bg=c(rep(c("yellow1", "indianred1", "g
reen1", "lightskyblue"),4)), spacing=0.2, ylim=c(min, max),
at=c(0.5,1.5,2.5,3.5, 5.5,6.5,7.5,8.5, 10.5,11.5,12.5,13.5, 15.5,16.5,17.5,18
.5), xaxt="n", cex=1.6, add=T)
axis(side=1, at=c(2, 7, 12, 17), labels=c("14 days", "28 days",
"42 days", "maturity"), mgp=c(2.5,1,0), cex.axis=1.6)
text(-0.4, 18.3, "a", font=2, cex=2)
legend(-0.1, 19.5, legend=c("Trout P.-High food", "Trout P.-Low food", "Wacis
sa R.-High food", "Wacissa R.-Low food"), pt.bg=c("yellow1", "indianred1", "g
reen1", "lightskyblue"),
```

```
col=c("black", "black"), bty="n", pch=21, cex=1.6, pt.cex=1.6, inset=c(0, -0.
02))
rect(0, 15.5, 5, 20)
points(c(0.5,1.5,2.5,3.5, 5.5,6.5,7.5,8.5, 10.5,11.5,12.5,13.5, 15.5,16.5,17.
5,18.5), tapply(dlong$SIZE, dlong$APF, mean), pch=24, bg="white", cex=2)
```

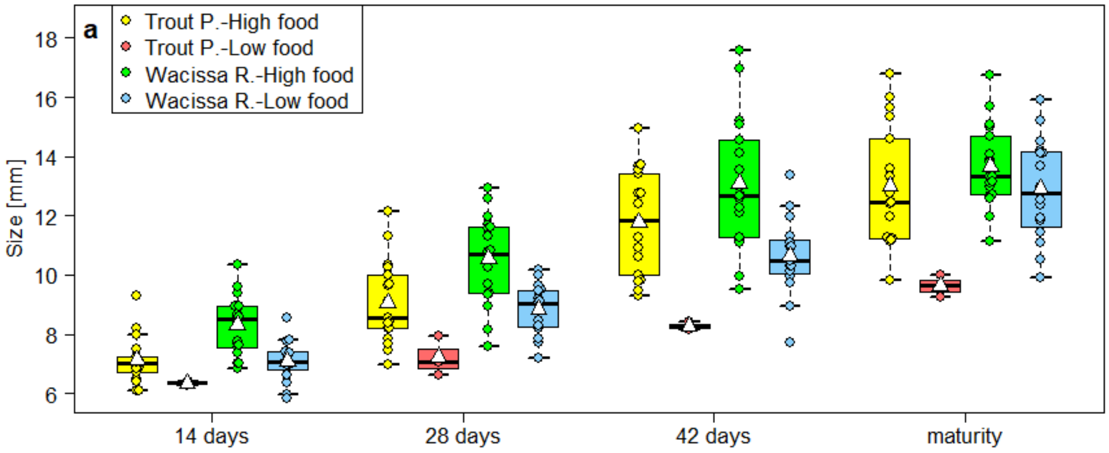


```
###

par(mfrow=c(1,2))
par(mar=c(4.5,4.5,1,1), xpd=F, cex=0.66)

# Figure 6b
boxplot(dlong$SIZE~dlong$AGESEX, xlab="", col=rep(c("lavenderblush1", "lavend
erblush4"), 4),
ylim=c(min, max), at=c(0.5,1.5,3,4,5.5,6.5,8,9), xaxt="n", ylab="Size [mm]",
las=1, cex.axis=1.6, cex.lab=1.6, xlim=c(0,9.5), outlier.color=NA, staplelwd=
2)
beeswarm(dlong$SIZE~dlong$AGESEX, pch=21, bg=c(rep(c("lavenderblush1", "laven
derblush4"),4)), spacing=0.2, ylim=c(min, max), at=c(0.5,1.5,3,4,5.5,6.5,8,9)
, xaxt="n", cex=1.6, add=T)
axis(side=1, at=c(1, 3.5, 6, 8.5), labels=c("14 days", "28 days",
"42 days", "maturity"), mgp=c(2.5,1,0), cex.axis=1.6)
text(0.1, 18.3, "b", font=2, cex=2)
legend(0.5, 19.5, legend=c("Female", "Male"), pt.bg=c("lavenderblush1", "lave
nderblush4"),
col=c("black", "black"), bty="n", pch=21, cex=1.6, pt.cex=1.6, inset=c(0, -0.
02))
rect(0.65, 17.2, 3.8, 20)
points(c(0.5,1.5,3,4,5.5,6.5,8,9), tapply(dlong$SIZE, dlong$AGESEX, mean), pc
h=24, bg="white", cex=2)

# Figure 6c
plot(dlong$SIZE~dlong$ISIZE, las=1, xlab="Size at birth [mm]", ylab="Size [mm
]", cex.lab=1.6, cex.axis=1.6, col="white", ylim=c(min, max))
points(dlong$SIZE[dlong$AGE %in% 1]~dlong$ISIZE[dlong$AGE %in% 1], pch=21, bg
```

```
="lightskyblue1", cex=1.6)
points(dlong$SIZE[dlong$AGE %in% 2]~dlong$ISIZE[dlong$AGE %in% 2], pch=21, bg
="green1", cex=1.6)
points(dlong$SIZE[dlong$AGE %in% 3]~dlong$ISIZE[dlong$AGE %in% 3], pch=21, bg
="yellow1", cex=1.6)
points(dlong$SIZE[dlong$AGE %in% 4]~dlong$ISIZE[dlong$AGE %in% 4], pch=21, bg
="indianred1", cex=1.6)
text(5.1, 18.3, "c", font=2, cex=2)
legend("bottomright", legend=c("14 days", "28 days", "42 days", "maturity"),
pt.bg=c("lightskyblue1", "green1", "yellow1", "indianred1"),
col=c("black", "black"), bty="n", pch=21, cex=1.6, pt.cex=1.6, inset=c(0, -0.
02))
rect(8.55, 5, 11, 9)
```

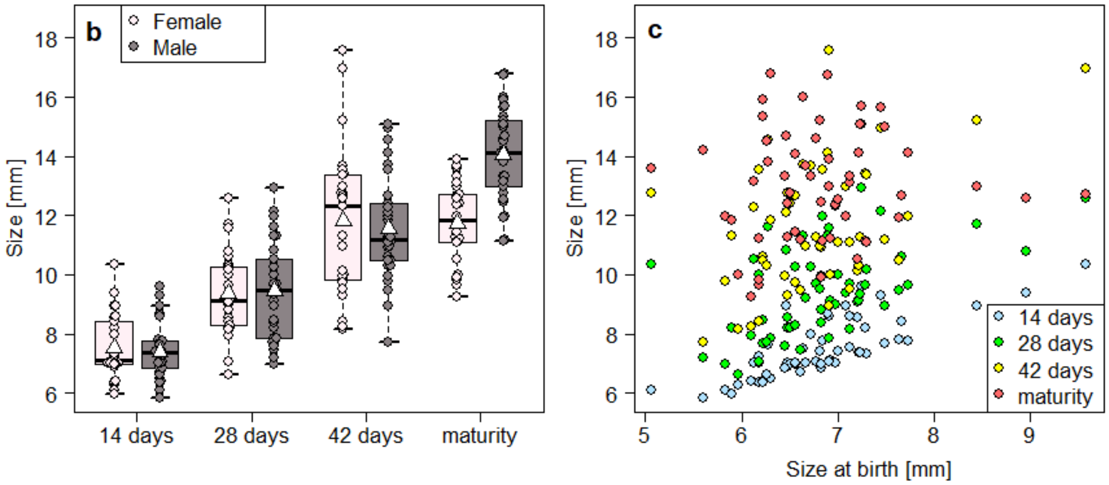

7. Analysis of age at sexual maturity

7.1 Distribution of age at sexual maturity

```
# exclude fish without data on their age at sexual maturity
dsub7<-d[is.na(d$AGEMAT)==F,]
nrow(dsub7) # 56 individuals

## [1] 56

# two-sided Kolmogorov-Smirnov test, showing no significant deviation from no
rmality
ks.test(dsub7$AGEMAT, pnorm, mean(dsub7$AGEMAT), sd(dsub7$AGEMAT))
##  One-sample Kolmogorov-Smirnov test
##
## data:  dsub7$AGEMAT
## D = 0.11177, p-value = 0.4862
## alternative hypothesis: two-sided

# histogram and quantile-quantile plot, also indicating that data are approxi
mately normally distributed
```

```
par(mfrow=c(1,2))
hist(dsub7$AGEMAT, breaks=50, main="", xlab="Age at sexual maturity [days]",
las=1)
qqnorm(dsub7$AGEMAT, pch = 1, frame = FALSE, main="", las=1)
qqline(dsub7$AGEMAT, col = "steelblue", lwd = 2)
```

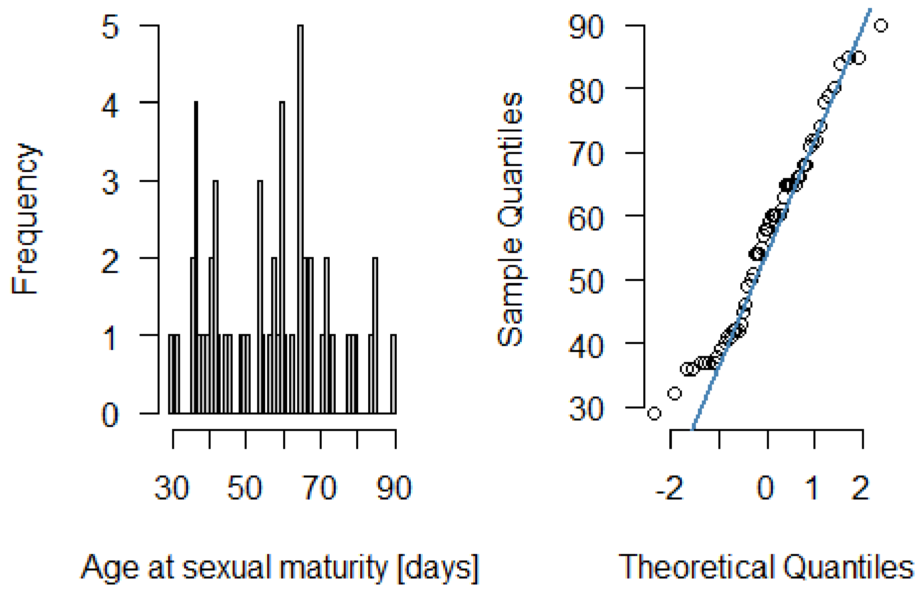

7.2 Computing proportional differences between populations, food levels and sexes

```
tapply(dsub7$AGEMAT, dsub7$LOCATION, mean)

##   Trout Wacissa
## 55.7619 57.0000

57.0000/55.76190

## [1] 1.022203

tapply(dsub7$AGEMAT, dsub7$FOOD, mean)

##     High      Low
## 51.22222 66.10000

66.10000/51.22222

## [1] 1.290456

tapply(dsub7$AGEMAT, dsub7$SEX, mean)
```

```
##   female     male  unknown 
## 47.74074 64.72414       NA

64.72414/47.74074

## [1] 1.355742

tapply(dsub7$AGEMAT, dsub7$SEX, sd)

##   female     male  unknown 
## 12.83503 13.30654       NA
```

7.3 Model 10: age at sexual maturity

```
# GLMM with Gaussian errors including the population of origin, experimental food level, size at birth, interaction between food level and size at birth, and sex as fixed effects, and maternal identity as a random effect
m10<-glmmTMB(AGEMAT ~ LOCATION + FOOD + ISIZE + FOOD:ISIZE + SEX + (1|MOM),
             family=gaussian, data=dsub7)
summary(m10)

##  Family: gaussian  ( identity )
## Formula:          
## AGEMAT ~ LOCATION + FOOD + ISIZE + FOOD:ISIZE + SEX + (1 | MOM)
## Data: dsub7
## 
##      AIC      BIC   logLik deviance df.resid 
##    416.9    433.1   -200.4    400.9       48 
## 
## Random effects:
## 
## Conditional model:
##  Groups   Name        Variance  Std.Dev.
##  MOM      (Intercept)  0.002905 0.0539  
##  Residual             75.223179 8.6731  
## Number of obs: 56, groups:  MOM, 27
## 
## Dispersion estimate for gaussian family (sigma^2): 75.2 
## 
## Conditional model:
##                  Estimate Std. Error z value Pr(>|z|)    
## (Intercept)        60.067     13.858   4.335 1.46e-05 ***
## LOCATIONWacissa    -2.649      2.928  -0.905  0.36568    
## FOODLow            71.501     24.945   2.866  0.00415 ** 
## ISIZE              -2.664      2.023  -1.317  0.18795    
## SEXmale            19.637      2.503   7.846 4.31e-15 ***
## FOODLow:ISIZE      -8.223      3.693  -2.227  0.02595 *  
## ---
## Signif. codes:  0 '***' 0.001 '**' 0.01 '*' 0.05 '.' 0.1 ' ' 1
```

```
# diagnostic plot of residuals versus fitted values
res_m10<-resid(m10)
fitted_m10<-fitted(m10)
plot(res_m10~fitted_m10, las=1)
```

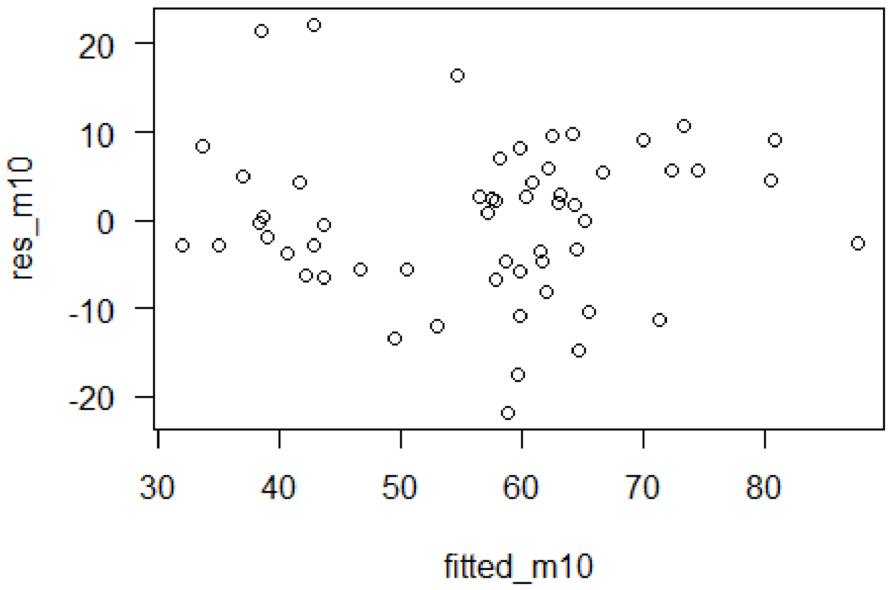


```
# obtaining a p-value for the effect of maternal identity
m10a<-glmmTMB(AGEMAT ~ LOCATION + FOOD + ISIZE + FOOD:ISIZE + SEX, family=gau
ssian, data=dsub7)
anova(m10, m10a)

## Data: dsub7
## Models:
## m10a: AGEMAT ~ LOCATION + FOOD + ISIZE + FOOD:ISIZE + SEX, zi=~0, disp=~1
## m10: AGEMAT ~ LOCATION + FOOD + ISIZE + FOOD:ISIZE + SEX + (1 | MOM), zi=~
0, disp=~1
##      Df    AIC    BIC  logLik deviance Chisq Chi Df Pr(>Chisq)
## m10a  7 414.87 429.05 -200.43   400.87
## m10   8 416.87 433.07 -200.43   400.87     0      1          1
```

7.4 Model 11: age at sexual maturity (test of influence of outliers)

This model excludes two fish with very early and one fish with very late age at maturity, identified as potential outliers in the QQ-plot.

```
# making a new dataset that only contains fish with an age at maturity betwee
n 33 and 89 days
dsub8<-dsub7[dsub7$AGEMAT > 32,]
```

```r
dsub8<-dsub8[dsub8$AGEMAT < 90,]
nrow(dsub8) # 53 individuals included in reduce dataset

## [1] 53

# GLMM using the subset of fish with age at maturity between 33 and 89 days,
including the same predictors as model 10
m11<-glmmTMB(AGEMAT ~ LOCATION + FOOD + ISIZE + FOOD:ISIZE + SEX + (1|MOM), f
amily=gaussian, data=dsub8)
summary(m11)

##  Family: gaussian  ( identity )
## Formula:
## AGEMAT ~ LOCATION + FOOD + ISIZE + FOOD:ISIZE + SEX + (1 | MOM)
## Data: dsub8
##
##      AIC      BIC   logLik deviance df.resid
##    396.7    412.4   -190.3    380.7       45
##
## Random effects:
##
## Conditional model:
##  Groups   Name        Variance  Std.Dev.
##  MOM      (Intercept) 2.634e-07 0.0005132
##  Residual             7.708e+01 8.7794148
## Number of obs: 53, groups:  MOM, 26
##
## Dispersion estimate for gaussian family (sigma^2): 77.1
##
## Conditional model:
##                  Estimate Std. Error z value Pr(>|z|)
## (Intercept)        54.146     17.270   3.135  0.00172 **
## LOCATIONWacissa    -3.003      2.988  -1.005  0.31478
## FOODLow            72.086     27.393   2.632  0.00850 **
## ISIZE              -1.684      2.574  -0.654  0.51296
## SEXmale            19.021      2.582   7.365 1.77e-13 ***
## FOODLow:ISIZE      -8.393      4.056  -2.069  0.03850 *
## ---
## Signif. codes:  0 '***' 0.001 '**' 0.01 '*' 0.05 '.' 0.1 ' ' 1

# diagnostic plot of residuals versus fitted values
res_m11<-resid(m11)
fitted_m11<-fitted(m11)
plot(res_m11~fitted_m11, las=1)
```

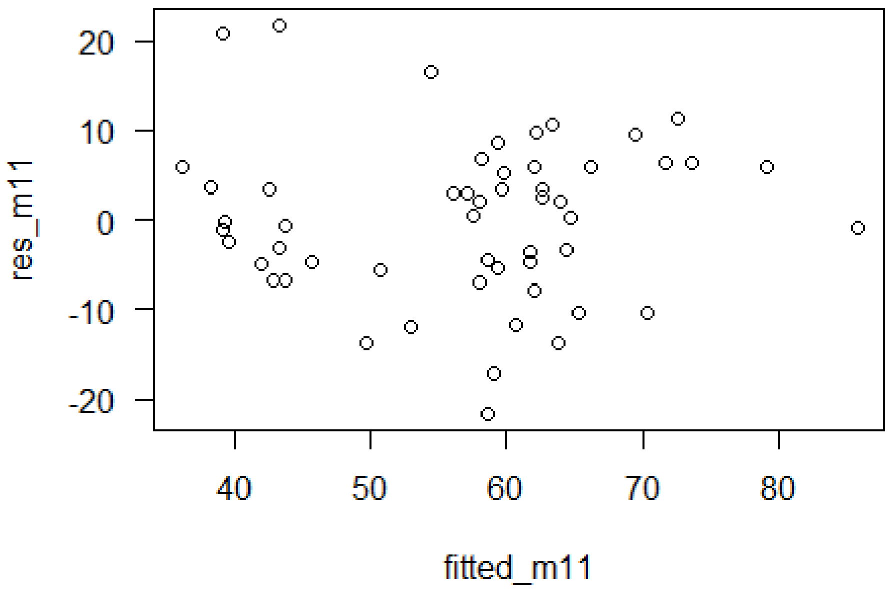


```
# obtaining a p-value for effect of maternal identity
m11a<-glmmTMB(AGEMAT ~ LOCATION + FOOD + ISIZE + FOOD:ISIZE + SEX, family=gau
ssian, data=dsub8)
anova(m11, m11a)

## Data: dsub8
## Models:
## m11a: AGEMAT ~ LOCATION + FOOD + ISIZE + FOOD:ISIZE + SEX, zi=~0, disp=~1
## m11: AGEMAT ~ LOCATION + FOOD + ISIZE + FOOD:ISIZE + SEX + (1 | MOM), zi=~
0, disp=~1
##      Df    AIC    BIC  logLik deviance Chisq Chi Df Pr(>Chisq)
## m11a  7 394.68 408.47 -190.34   380.68
## m11   8 396.68 412.45 -190.34   380.68     0      1          1
```

7.5 Correlation between age at maturity and other variables

```
# correlation with maternal size
cor.test(dsub7$AGEMAT, dsub7$MOMSZ)

##
##  Pearson's product-moment correlation
##
## data:  dsub7$AGEMAT and dsub7$MOMSZ
## t = -0.29903, df = 54, p-value = 0.7661
## alternative hypothesis: true correlation is not equal to 0
## 95 percent confidence interval:
##  -0.3003486  0.2246432
```

```
## sample estimates:
##         cor
## -0.04065858

# correlation with size at maturity
cor.test(dsub7$AGEMAT, dsub7$SMAT)

##
##  Pearson's product-moment correlation
##
## data:  dsub7$AGEMAT and dsub7$SMAT
## t = 1.3807, df = 54, p-value = 0.1731
## alternative hypothesis: true correlation is not equal to 0
## 95 percent confidence interval:
##  -0.0822385  0.4268339
## sample estimates:
##       cor
## 0.1846544
```

7.6 Difference in age at maturity between fish born large or small as a function of food level

```
# mean age at maturity of low-food fish with a size at birth of < 6.5mm and >
7mm, respectively
mean(dsub7$AGEMAT[dsub7$ISIZE < 6.50 & dsub7$FOOD %in% "Low"]) # 71.625

## [1] 71.625

mean(dsub7$AGEMAT[dsub7$ISIZE > 7.00 & dsub7$FOOD %in% "Low"]) # 55.2

## [1] 55.2

71.625-55.2 # 16.425

## [1] 16.425

# mean age at maturity of high-food fish with a size at birth of < 6.5mm and
> 7mm, respectively
mean(dsub7$AGEMAT[dsub7$ISIZE < 6.50 & dsub7$FOOD %in% "High"]) # 56.75

## [1] 56.75

mean(dsub7$AGEMAT[dsub7$ISIZE > 7.00 & dsub7$FOOD %in% "High"]) # 46.30769

## [1] 46.30769

56.75-46.30769 # 10.44231

## [1] 10.44231
```

7.7 Figure 7

```
par(mfrow=c(1,3))
par(mar=c(4.5,4.5,1,1), xpd=T)

# Figure 7a
plot(dsub7$AGEMAT~dsub7$FOOD, las=1, xlab="Food level", ylab="", pch=21, outl
ier.color=NA, cex.lab=1.6, cex.axis=1.6, xaxt="n")
axis(1, at=c(1,2), labels=c("High", "Low"), mgp=c(2.5,1,0), cex.axis=1.6)
title(ylab="Age at sexual maturity [days]", mgp=c(2.7,2.5,0), cex.lab=1.6)
beeswarm(dsub7$AGEMAT~dsub7$FOOD, bg="lavenderblush4", cex=1.6, pch=21, add=T
)
text(0.6,90, "a", font=2, cex=2)
points(tapply(dsub7$AGEMAT, dsub7$FOOD, mean), pch=24, bg="white", cex=2.5)

# Figure 7b
plot(dsub7$AGEMAT~dsub7$LOCATION, las=1, xlab="Population", ylab="", pch=21,
outlier.color=NA, cex.lab=1.6, cex.axis=1.6, xaxt="n")
axis(1, at=c(1,2), labels=c("Trout P.", "Wacissa R."), mgp=c(2.5,1,0), cex.ax
is=1.6)
title(ylab="Age at sexual maturity [days]", mgp=c(2.7,2.5,0), cex.lab=1.6)
beeswarm(dsub7$AGEMAT~dsub7$LOCATION, bg="lavenderblush4", cex=1.6, pch=21, a
dd=T)
text(0.6,90, "b", font=2, cex=2)
points(tapply(dsub7$AGEMAT, dsub7$LOCATION, mean), pch=24, bg="white", cex=2.
5)

# Figure 7c
dsub7$SEX<-factor(dsub7$SEX) # remove empty level "unknown" from column "SEX"
plot(dsub7$AGEMAT~dsub7$SEX, las=1, xlab="Sex", ylab="", pch=21, outlier.colo
r=NA, cex.lab=1.6, cex.axis=1.6, xaxt="n")
axis(1, at=c(1,2), labels=c("Female", "Male"), mgp=c(2.5,1,0), cex.axis=1.6)
title(ylab="Age at sexual maturity [days]", mgp=c(2.7,2.5,0), cex.lab=1.6)
beeswarm(dsub7$AGEMAT~dsub7$SEX, bg="lavenderblush4", cex=1.6, pch=21, add=T)
text(0.6,90, "c", font=2, cex=2)
points(tapply(dsub7$AGEMAT, dsub7$SEX, mean), pch=24, bg="white", cex=2.5)
```

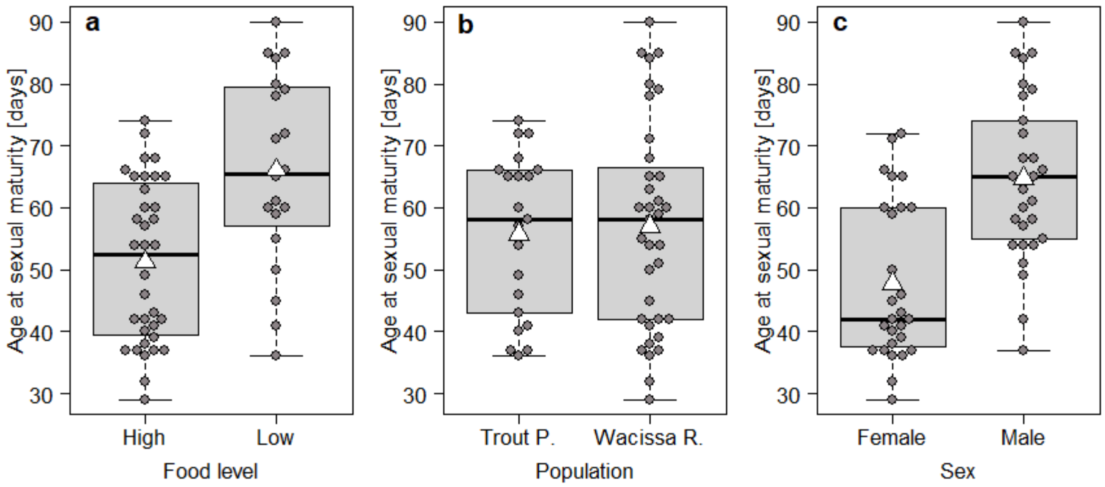


```
###

# save partial regression coefficients for Figure 7d
m10.coef<-as.data.frame(summary(m10)$coefficients[1])
m10_inter<-m10.coef[1,1]
m10_locWR<-m10.coef[2,1]
m10_foodL<-m10.coef[3,1]
m10_isize<-m10.coef[4,1]
m10_sexMA<-m10.coef[5,1]
m10_FLisi<-m10.coef[6,1]

# save ranges of size at birth for high- and low-food fish, for Figure 7d
isi.H<-seq(min(dsub7$ISIZE[dsub7$FOOD %in% "High"]), max(dsub7$ISIZE[dsub7$FO
OD %in% "High"]), 0.1)
isi.L<-seq(min(dsub7$ISIZE[dsub7$FOOD %in% "Low"]), max(dsub7$ISIZE[dsub7$FOO
D %in% "Low"]), 0.1)

# save proportion of WR fish and proportion of males, for Figure 7d
propWR<-nrow(dsub7[dsub7$LOCATION %in% "Wacissa",])/nrow(dsub7)
propMA<-nrow(dsub7[dsub7$SEX %in% "male",])/nrow(dsub7)

par(mfrow=c(1,3))
par(mar=c(4.5,4.5,1,1), xpd=F)

# Figure 7d
plot(dsub7$AGEMAT~dsub7$ISIZE, las=1, xlab="Size at birth [mm]", ylab="", cex
.lab=1.6, cex.axis=1.6, col="white")
title(ylab="Age at sexual maturity [days]", mgp=c(2.7,2.5,0), cex.lab=1.6)
text(5.2,90, "d", font=2, cex=2)

points(dsub7$AGEMAT[dsub7$FOOD %in% "High"]~dsub7$ISIZE[dsub7$FOOD %in% "High
"], pch=21, bg="yellow1", cex=1.6)
points(dsub7$AGEMAT[dsub7$FOOD %in% "Low"]~dsub7$ISIZE[dsub7$FOOD %in% "Low"]
, pch=21, bg="indianred1", cex=1.6)
```

```
lines(isi.H, m10_inter + m10_locWR*propWR + m10_foodL*0 + m10_isize*isi.H + m
10_sexMA*propMA + m10_FLisi*0*isi.H, lwd=1.8, col="yellow4")
lines(isi.L, m10_inter + m10_locWR*propWR + m10_foodL*1 + m10_isize*isi.L + m
10_sexMA*propMA + m10_FLisi*1*isi.L, lwd=1.8, col="indianred3")

legend("topright", legend=c("High food", "Low food"), pt.bg=c("yellow1", "ind
ianred1"),
col=c("black", "black"), bty="n", pch=21, cex=1.6, pt.cex=1.6, inset=c(0, -0.
02))
rect(7.45, 82, 10, 93)

# Figure 7e
plot(dsub7$AGEMAT~dsub7$MOMSZ, las=1, xlab="Mother's size [mm]", ylab="", cex
.lab=1.6, cex.axis=1.6, xlim=c(18, 33), xaxt="n", cex=1.6)
axis(1, at=c(18, 22, 26, 30), cex.axis=1.6, las=1)
title(ylab="Age at sexual maturity [days]", mgp=c(2.7,2.5,0), cex.lab=1.6)
text(18.5, 90, "e", font=2, cex=2)

# Figure 7f
plot(dsub7$AGEMAT~dsub7$SMAT, las=1, xlab="Size at sexual maturity [mm]", yla
b="", cex.lab=1.6, cex.axis=1.6,
xlim=c(9,18), cex=1.6)
title(ylab="Age at sexual maturity [days]", mgp=c(2.7,2.5,0), cex.lab=1.6)
text(9.4,90, "f", font=2, cex=2)
```

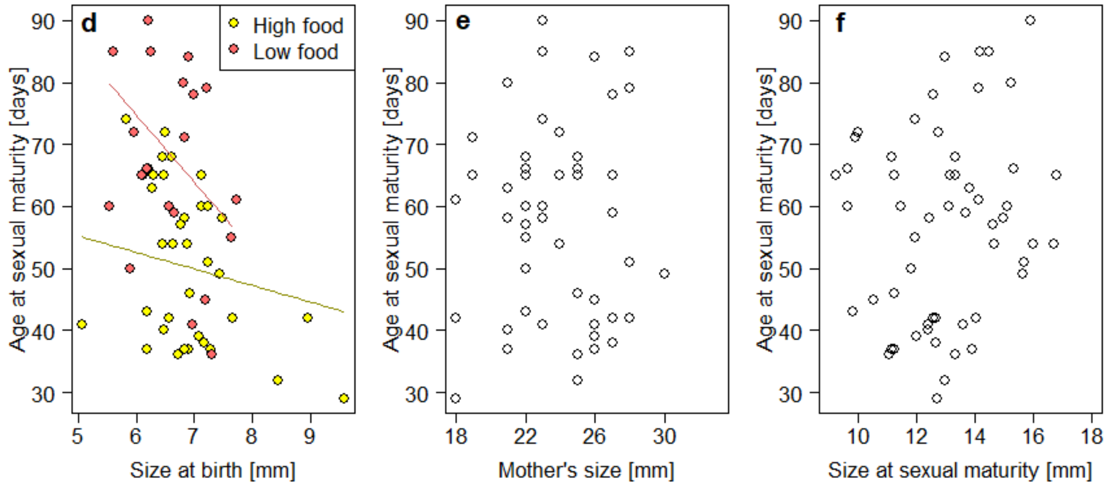

Supplement: Supplementary file 1 — Appendix S1 [file ECE3-11-6391-s001.pdf]
